# Postoperative outcomes after minimally invasive esophagectomy: an international cohort study from the Oesophagogastric Anastomosis Audit (OGAA)

**DOI:** 10.1186/s12893-025-02941-6

**Published:** 2025-05-22

**Authors:** Oesophago-Gastric Anastomotic Audit (OGAA) Collaborative, D. Alderson, J. Bundred, R. P. T. Evans, J. Gossage, E. A. Griffiths, B. Jefferies, S. K. Kamarajah, S. McKay, I. Mohamed, D. Nepogodiev, K Siaw-Acheampong, P. Singh, R. van Hillegersberg, R. Vohra, K. Wanigasooriya, T. Whitehouse, A. Gjata, J. I. Moreno, F. R. Takeda, B. Kidane, R Guevara Castro, T. Harustiak, A. Bekele, A. Kechagias, I. Gockel, A. Kennedy, A. Da Roit, A. Bagajevas, J. S. Azagra, H. A. Mahendran, L. Mejía-Fernández, B. P. L. Wijnhoven, J El Kafsi, R. H. Sayyed, M. Sousa, A. S. Sampaio, I. Negoi, R. Blanco, B. Wallner, P. M. Schneider, P. K. Hsu, A. Isik, S. Gananadha, V. Wills, M. Devadas, C. Duong, M. Talbot, M. W. Hii, R. Jacobs, N. A. Andreollo, B. Johnston, G. Darling, A. Isaza-Restrepo, G. Rosero, F Arias-Amézquita, D. Raptis, J. Gaedcke, D. Reim, J. Izbicki, J. H. Egberts, S. Dikinis, D. W. Kjaer, M. H. Larsen, M. P. Achiam, J. Saarnio, D. Theodorou, T. Liakakos, D. P. Korkolis, W. B. Robb, C. Collins, T. Murphy, J. Reynolds, V. Tonini, M. Migliore, L. Bonavina, M. Valmasoni, R. Bardini, J. Weindelmayer, M. Terashima, R. E. White, E. Alghunaim, M. Elhadi, A. M. Leon-Takahashi, H. Medina-Franco, P. C. Lau, K. E. Okonta, J. Heisterkamp, C. Rosman, R. van Hillegersberg, G. Beban, R. Babor, A. Gordon, J. I. Rossaak, K. M. I. Pal, A. U. Qureshi, S. A. Naqi, A. A. Syed, J. Barbosa, C. S. Vicente, J. Leite, J. Freire, R. Casaca, R. C. T. Costa, R. R. Scurtu, S. S. Mogoanta, C. Bolca, S. Constantinoiu, D. Sekhniaidze, M. Bjelović, J. B. Y. So, G. Gačevski, C. Loureiro, M. Pera, A. Bianchi, M Moreno Gijón, J Martín Fernández, MS Trugeda Carrera, M. Vallve-Bernal, MA Cítores Pascual, S. Elmahi, I. Halldestam, J. Hedberg, S. Mönig, S. Gutknecht, M. Tez, A. Guner, M. B. Tirnaksiz, E. Colak, B. Sevinç, A. Hindmarsh, I. Khan, D. Khoo, R. Byrom, J. Gokhale, P. Wilkerson, P. Jain, D. Chan, K. Robertson, S. Iftikhar, R. Skipworth, M. Forshaw, S. Higgs, J. Gossage, R. Nijjar, Y. K. S. Viswanath, P. Turner, S. Dexter, A. Boddy, W. H. Allum, S. Oglesby, E. Cheong, D. Beardsmore, R. Vohra, N. Maynard, R. Berrisford, S. Mercer, S. Puig, R. Melhado, C. Kelty, T. Underwood, K. Dawas, W. Lewis, A. Al-Bahrani, G. Bryce, M. Thomas, A. T. Arndt, F. Palazzo, R. A. Meguid, J. Fergusson, E. Beenen, C. Mosse, J. Salim, S. Cheah, T. Wright, M. P. Cerdeira, P. McQuillan, M. Richardson, H. Liem, J. Spillane, M. Yacob, F. Albadawi, T. Thorpe, A. Dingle, C. Cabalag, K. Loi, O. M. Fisher, S. Ward, M. Read, M. Johnson, R. Bassari, H. Bui, I. Cecconello, R. A. A. Sallum, J. R. M. da Rocha, L. R. Lopes, V. Tercioti, J. D. S. Coelho, J. A. P. Ferrer, G. Buduhan, L. Tan, S. Srinathan, P. Shea, J. Yeung, F. Allison, P. Carroll, F. Vargas-Barato, F. Gonzalez, J. Ortega, L. Nino-Torres, T. C. Beltrán-García, L. Castilla, M. Pineda, A. Bastidas, J. Gómez-Mayorga, N. Cortés, C. Cetares, S. Caceres, S. Duarte, A. Pazdro, M. Snajdauf, H. Faltova, M. Sevcikova, P. B. Mortensen, N. Katballe, T. Ingemann, B. Morten, I. Kruhlikava, A. P. Ainswort, N. M. Stilling, J. Eckardt, J. Holm, M. Thorsteinsson, M. Siemsen, B. Brandt, B. Nega, E. Teferra, A. Tizazu, J. H. Kauppila, V. Koivukangas, S. Meriläinen, R. Gruetzmann, C. Krautz, G. Weber, H. Golcher, G. Emons, A. Azizian, M. Ebeling, S. Niebisch, N. Kreuser, G. Albanese, J. Hesse, L. Volovnik, U. Boecher, M. Reeh, S. Triantafyllou, D. Schizas, A. Michalinos, E. Balli, M. Mpoura, A. Charalabopoulos, D. K. Manatakis, D. Balalis, J. Bolger, C. Baban, A. Mastrosimone, O. McAnena, A. Quinn, C. B. Ó Súilleabháin, M. M. Hennessy, I. Ivanovski, H. Khizer, N. Ravi, N. Donlon, M. Cervellera, S. Vaccari, S. Bianchini, l Sartarelli, E. Asti, D. Bernardi, S. Merigliano, L. Provenzano, M. Scarpa, L. Saadeh, B. Salmaso, G. De Manzoni, S. Giacopuzzi, R. La Mendola, C. A. De Pasqual, Y. Tsubosa, M. Niihara, T. Irino, R. Makuuchi, K. Ishii, M. Mwachiro, A. Fekadu, A. Odera, E. Mwachiro, D. AlShehab, H. A. Ahmed, A. O. Shebani, A. Elhadi, F. A. Elnagar, H. F. Elnagar, S. T. Makkai-Popa, L. F. Wong, Y. R. Tan, S. Thannimalai, C. A. Ho, W. S. Pang, J. H. Tan, H. N. L. Basave, R. Cortés-González, S. M. Lagarde, J. J. B. van Lanschot, C. Cords, W. A. Jansen, I. Martijnse, R. Matthijsen, S. Bouwense, B. Klarenbeek, M. Verstegen, F. van Workum, J. P. Ruurda, P. C. van der Sluis, M. de Maat, N. Evenett, P. Johnston, R. Patel, A. MacCormick, M. Young, B. Smith, C. Ekwunife, A. H. Memon, K. Shaikh, A. Wajid, N. Khalil, M. Haris, Z. U. Mirza, S. B. A. Qudus, M. Z. Sarwar, A. Shehzadi, A. Raza, M. H. Jhanzaib, J. Farmanali, Z. Zakir, O. Shakeel, I. Nasir, S. Khattak, M. MA BaigNoor, H. H. Ahmed, A. Naeem, A. C. Pinho, R. da Silva, A. Bernardes, J. C. Campos, H. Matos, T. Braga, C. Monteiro, P. Ramos, F. Cabral, M. P. Gomes, P. C. Martins, A. M. Correia, J. F. Videira, C. Ciuce, R. Drasovean, R. Apostu, C. Ciuce, S. Paitici, A. E. Racu, C. V. Obleaga, M. Beuran, B. Stoica, C. Ciubotaru, V. Negoita, I. Cordos, R. D. Birla, D. Predescu, P. A. Hoara, R. Tomsa, V. Shneider, M. Agasiev, I. Ganjara, D. Gunjić, M. Veselinović, T. Babič, T. S. Chin, A. Shabbir, G. Kim, I Díez del Val, S. Leturio, J. M. Ramón, M Dal Cero, S. Rifá, M. Rico, A Pagan Pomar, J. A. Martinez Corcoles, J. L. Rodicio Miravalles, S. A. Pais, S. A. Turienzo, L. S. Alvarez, P. V. Campos, A. G. Rendo, S. S. García, E. P. G. Santos, E. T. Martínez, M. J. Fernández Díaz, C Magadán Álvarez, V Concepción Martín, C Díaz López, A Rosat Rodrigo, L. E. Pérez Sánchez, M Bailón Cuadrado, C Tinoco Carrasco, E Choolani Bhojwani, D. P. Sánchez, M. E. Ahmed, T. Dzhendov, F. Lindberg, M. Rutegård, M. Sundbom, C. Mickael, N. Colucci, A. Schnider, S. Er, E. Kurnaz, S. Turkyilmaz, A. Turkyilmaz, R. Yildirim, B. E. Baki, N. Akkapulu, O. Karahan, N. Damburaci, R. Hardwick, P. Safranek, V. Sujendran, J. Bennett, Z. Afzal, M. Shrotri, B. Chan, K. Exarchou, T. Gilbert, T. Amalesh, D. Mukherjee, S. Mukherjee, T. H. Wiggins, R. Kennedy, S. McCain, A. Harris, G. Dobson, N. Davies, I. Wilson, D. Mayo, D. Bennett, R. Young, P. Manby, N. Blencowe, M. Schiller, B. Byrne, D. Mitton, V. Wong, A. Elshaer, M. Cowen, V. Menon, L. C. Tan, E. McLaughlin, R. Koshy, C. Sharp, H. Brewer, N. Das, M. Cox, W. Al Khyatt, D. Worku, R. Iqbal, L. Walls, R. McGregor, G. Fullarton, A. Macdonald, C. MacKay, C. Craig, S. Dwerryhouse, S. Hornby, S. Jaunoo, M. Wadley, C. Baker, M. Saad, M. Kelly, A. Davies, F. Di Maggio, S. McKay, P. Mistry, R. Singhal, O. Tucker, S. Kapoulas, S. Powell-Brett, P. Davis, G. Bromley, L. Watson, R. Verma, J. Ward, V. Shetty, C. Ball, K. Pursnani, A. Sarela, H Sue Ling, S. Mehta, J. Hayden, N. To, T. Palser, D. Hunter, K. Supramaniam, Z. Butt, A. Ahmed, S. Kumar, A. Chaudry, O. Moussa, A. Kordzadeh, B. Lorenzi, M. Wilson, P. Patil, I. Noaman, J. Willem, G. Bouras, R. Evans, M. Singh, H. Warrilow, A. Ahmad, N. Tewari, F. Yanni, J. Couch, E. Theophilidou, J. J. Reilly, P. Singh, Gijs van Boxel, K. Akbari, D. Zanotti, B. Sgromo, G. Sanders, T. Wheatley, A. Ariyarathenam, A. Reece-Smith, L. Humphreys, C. Choh, N. Carter, B. Knight, P. Pucher, A. Athanasiou, I. Mohamed, B. Tan, M. Abdulrahman, J. Vickers, K. Akhtar, R. Chaparala, R. Brown, M. M. A. Alasmar, R. Ackroyd, K. Patel, A. Tamhankar, A. Wyman, R. Walker, B. Grace, N. Abbassi, N. Slim, L. Ioannidi, G. Blackshaw, T. Havard, X. Escofet, A. Powell, A. Owera, F. Rashid, P. Jambulingam, J. Padickakudi, H. Ben-Younes, K. Mccormack, I. A. Makey, M. K. Karush, C. W. Seder, M. J. Liptay, G. Chmielewski, E. L. Rosato, A. C. Berger, R. Zheng, E. Okolo, A. Singh, C. D. Scott, M. J. Weyant, J. D. Mitchell

**Affiliations:** Department of Applied Health Sciences, School of Applied Health Sciences, College of Medicine and Health, University of Birmingham, Birmingham, B15 2 TH UK

**Keywords:** Minimally invasive, Esophagectomy, Outcomes, Pulmonary complications

## Abstract

**Objective:**

To compare the postoperative pulmonary complications (PPC) after minimally invasive or open transthoracic esophagectomy for esophageal cancer in an international, multicenter cohort.

**Summary of background data:**

Ongoing debate exists around the optimal surgical approach for esophageal cancer, with limited data assessing the external validity of randomised trials on outcomes of MIE

**Methods:**

Patients undergoing open (OE, *n* = 744), hybrid (HE, *n* = 500), and totally minimally invasive esophagectomy (TMIE, *n* = 540) for esophageal cancer were identified from the international, prospective Oesophagogastric Anastomosis Audit (OGAA). Multivariable models were used to investigate PPC (primary outcome) as well as overall complications, major complications, anastomotic leak and 90-day mortality (secondary outcomes).

**Results:**

PPC rates were lower after TMIE compared to OE and HE (28% vs 37% vs 39%, *p* = 0.002), even on adjusted analyses compared to OE (odds ratio (OR): 0.60, CI_95%_: 0.45—0.78). TMIE was also associated with significantly lower overall complications (OR: 0.68, CI_95%_: 0.52—0.88) compared to OE, but not for major complications (OR: 0.90, CI_95%_: 0.67—1.21), anastomotic leak (OR: 1.39, CI_95%_: 0.96—2.01) and 90-day mortality (OR: 0.49, CI_95%_: 0.22—1.04). Sensitivity analyses by underlying respiratory disease, neoadjuvant chemoradiotherapy or high-volume centers confirmed above findings.

**Conclusion:**

This study provides real-world data that TMIE was associated with lower 90-day PPC than OE and HE approaches, especially in patients with underlying respiratory disease or receiving neoadjuvant chemoradiotherapy. These warrant a further review into causes and mechanisms in selected patients, and that quality assurance in delivery of TMIE is probably of major importance. The ideal surgical approach remains unclear, and ongoing trials will provide more evidence within a few years that may clarify the optimum approach to locally advanced esophageal cancers.

**Supplementary Information:**

The online version contains supplementary material available at 10.1186/s12893-025-02941-6.

## Introduction

Multimodality treatment with neoadjuvant therapy and esophagectomy remains the curative treatment of patients with resectable esophageal cancer [1, 2]. Although there has been substantive improvement in postoperative mortality after esophagectomies, morbidity rates remain as high as 70% [3] and patients are associated with reduced quality of life [4, 5]. Furthermore, the detrimental impact of perioperative complications on long-term survival is also well understood [6–8]. To improve perioperative morbidity, there has been a dramatic increase in the adoption of minimally invasive esophagectomy (MIE), through implementation of these programs in centers [9].

There are several approaches used for esophagectomies such as Ivor-Lewis, McKeown, and Transhiatal. Therefore, this warrants dedicated evaluation on the role of MIE techniques. However, the benefits of MIE in patients undergoing transthoracic esophagectomy remains unclear. Firstly, evidence have until recently been limited to two randomised clinical trials [10, 11]. Both these trials only compared either totally MIE (TMIE) or hybrid MIE with open esophagectomy demonstrating significantly lower rates of postoperative pulmonary complications (PPC) compared to open esophagectomy. However, adoption of these MIE techniques into routine clinical practice were associated with higher overall and pulmonary complications and reoperation rate [12, 13]. Secondly, present studies are limited to cohort studies either from selected high-volume centers, single center or national series. Therefore, global data, including low- and middle-income countries comparing MIE and open surgery are needed to assess its impact on both postoperative complications.

The primary aim of the present study was to compare the incidence of PPC between OE, HE and TMIE using data from the Oesophago-Gastric Anastomosis Audit (OGAA). The secondary aims were to assess the rates of postoperative morbidity (i.e., overall, and major complications, anastomotic leaks, and 90-day mortality).

## Methods

### Study design and setting

This study is a secondary pre-planned analysis of the OGAA cohort study. The OGAA cohort study was an international multicenter prospective study including 137 centers across 41 countries [14–16]. All hospitals performing esophagectomies for esophageal cancers were eligible to be included in this cohort study. Patient-level data were collected over a nine-month period from 1 st April 2018 to 31 st December 2018 with follow-up of all patients up to 90-days after surgery. This study was delivered using a collaborative model, which has been described previously. This methodology and process has been successful in delivering several international and national cohort studies [15–17]. Briefly, a consultant or attending surgeon supervised data collection at each hospital, together with a team of clinicians, ensuring that it was performed in accordance with a pre-specified protocol. The study was conducted according to STROBE guidelines for observational studies [18].

### Ethics and reporting

Ethical approval was dependent on local protocols, and was country-specific, as previously described [15, 16]. Ongoing study approval was maintained locally throughout the duration of the study.

### Inclusion and exclusion criteria

During the pre-defined data collection period, all consecutive adult patients undergoing elective (planned) curative esophagectomy for esophageal cancers (i.e. adenocarcinoma and SCC) were included. For the present analysis, patients undergoing two-stage transthoracic esophagectomy using any combination of open, robotic or standard minimal access approaches were included. Exclusion criteria were: (i) extended total gastrectomy, transhiatal esophagectomy, three-stage (i.e. McKeown) esophagectomy or left thoracoabdominal; (ii) pharyngolaryngo-oesophagectomy; (iii) colonic interposition or small bowel jejunal interposition reconstruction; (iv) emergency resection; and (v) resection for benign disease, as previously described [19, 20].

### Outcome measures

The primary outcome measure was postoperative pulmonary complications, according to the Esophagectomy Complications Consensus Group [21, 22]. Postoperative pulmonary complications were defined as pneumonia, pleural effusion requiring additional drainage procedure, pneumothorax requiring treatment, atelectasis mucous plugging requiring bronchoscopy, respiratory failure requiring reintubation, acute respiratory distress syndrome, acute aspiration, tracheobronchial injury and chest tube maintenance for air leak for > 10 days postoperatively. Secondary outcome measures were overall complications, major complications, anastomotic leaks, and 90-day mortality. Major postoperative complications were defined as those of Clavien-Dindo Grade III-V [20]. Anastomotic leaks were defined full thickness GI defect involving esophagus, anastomosis, staple line, or conduit irrespective of presentation or method of identification according to the ECCG [21]. Broadly, these leaks were defined as type I, type II or type III anastomotic leaks as defined according to the ECCG guidance.

### Data collection

The process of data collection was based on case report forms (CRFs), which were for data recording only. Detailed reporting on data collection process has been reported elsewhere [23–25].

### Definitions of variables

A range of patient-, tumour- and treatment-related factors were assessed. Data for a range of center-specific variables were also collected. Country income was defined as high-income (HIC) or low- or middle-income countries (LMIC), according to the World Bank Data [26]. The center volume [27, 28] was based on the number of cases treated by each center during the study period (nine months), from which the number of cases per year was estimated. The resulting variable was then categorized for analysis, based on tertiles, such that there were approximately equal numbers of patients in each category. The resulting categories were < 28 (*n* = 94 centers; HIC—68, LMIC—26), 28–50 (*n* = 28 centers; HIC—25, LMIC—3) and > 50 cases (*n* = 15 centers, HIC—13, LMIC—2) per year. The TNM staging was based on pathology and used the 8 th edition definitions [29].

### Statistical analysis

Continuous variables that were normally distributed were reported as mean ± standard deviation (SD), with *p*-values from independent sample t-tests. For continuous, and non-normally distributed data, we reported them as medians and interquartile ranges (IQRs). Categorical ordinal variables were also assessed using Mann–Whitney U-tests, whilst nominal variables were analysed using Fisher’s exact-tests or Chi^2^-tests, for variables with two or more than two categories, respectively. Multilevel multivariable analyses were then performed, to produce adjusted odds ratio (OR) on surgical techniques on outcomes to account for the multi-level structure of the data, by adjusting for within-centre correlations of outcomes. As such, the centre was set as the subject effect, and the patient ID as the within-subject effect, with an exchangeable correlation structure assumed. For all models, a range of centre-, patient-, tumour- and treatment-related factors were assessed. Variables included in the multivariable models were selected based on clinical relevance, existing literature, and availability in the dataset. The adjusted model includes center- (center volume, country income), patient- (i.e. age, gender, ASA grade, Charlson comorbidity index, smoking status and body mass index), tumour- (i.e. tumor histology, tumor location, AJCC clinical T and N classification) and treatment-related (i.e. preoperative nutrition, neoadjuvant therapy, surgical approach, anastomosis site) factors. Although our models were not intended for prognostic prediction, we report the c-statistic (area under the curve) for each outcome model as an indicator of overall model performance. Sensitivity analyses were performed by patients with respiratory disease, neoadjuvant chemoradiotherapy and high-volume centers. A *p*-value of < 0.05 was considered statistically significant and 2-sided 5% significance level was applied. Data analysis was performed using R Foundation Statistical software (R 3.2.2) with TableOne, ggplot2, Hmisc, and finalfit packages (R Foundation for Statistical Computing, Vienna, Austria) as previously described [15, 16].

## Results

### Clinicopathologic characteristics

**Fig. 1:**
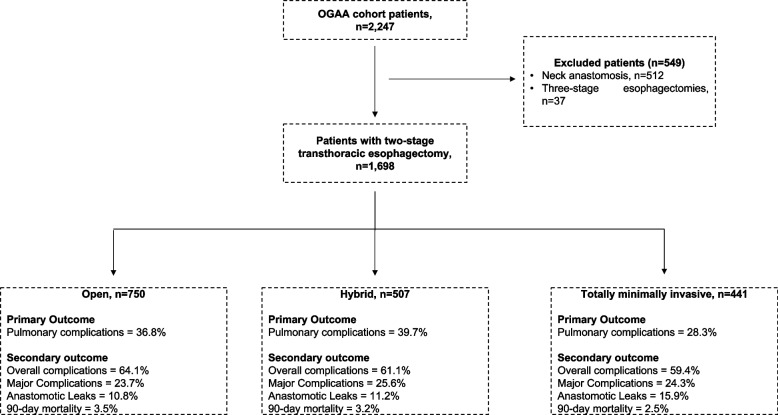
Flow chart on inclusion of patients with esophageal cancers (i.e. adenocarcinoma and squamous cell carcinoma) from the Oesophagogastric Anastomosis Audit

Of the 2,247 patients identified from the OGAA study, 1,784 patients received transthoracic OE (*n* = 744), HE (*n* = 500) or TMIE (*n* = 540) for esophageal cancers (Fig. 1). Baseline characteristics of patients are presented in Tables 1 and 2. Patients receiving TMIE were more likely to be from high volume (< 50 cases/year) (35% vs 27%, *p* < 0.001) and from high-income countries (96% vs 93%, *p* < 0.001) than patients receiving OE.

**Table 1:** Hospital- and patient-level clinicopathologic characteristics of patients with esophageal cancers receiving open, hybrid and totally minimally invasive esophagectomy

		**Open,** ***n* = 750**	**Hybrid,** ***n* = 507**	**Totally minimally invasive,** ***n* = 441**	***p*-value**
Hospital-level factors					
Center volume	< 28	232 (30.9)	129 (25.4)	155 (35.1)	< 0.001
	28–50	313 (41.7)	212 (41.8)	130 (29.5)	
	≥ 51	205 (27.3)	166 (32.7)	156 (35.4)	
Country income	High income country	696 (92.8)	498 (98.2)	424 (96.1)	< 0.001
	Low-Middle income country	54 (7.2)	9 (1.8)	17 (3.9)	
Patient-level factors					
Age at surgery		63.9 (10.1)	66.0 (9.6)	64.7 (9.8)	0.001
Sex	Female	155 (20.7)	89 (17.6)	74 (16.8)	0.182
	Male	595 (79.3)	418 (82.4)	367 (83.2)	
ASA Grade	1	108 (14.4)	72 (14.2)	38 (8.6)	< 0.001
	2	423 (56.4)	284 (56.0)	225 (51.0)	
	3–4	219 (29.2)	151 (29.8)	178 (40.4)	
Smoking status	Never smoker	300 (40.0)	181 (35.7)	142 (32.2)	0.007
	Ex-smoker	320 (42.7)	234 (46.2)	216 (49.0)	
	Current smoker	100 (13.3)	79 (15.6)	77 (17.5)	
	Unknown	30 (4.0)	13 (2.6)	6 (1.4)	
Respiratory Disease	No	660 (88.0)	457 (90.1)	377 (85.5)	0.090
	Yes	90 (12.0)	50 (9.9)	64 (14.5)	
Body mass index	≤ 18.5	32 (4.3)	13 (2.6)	8 (1.8)	0.020
	18.6–24.9	272 (36.3)	187 (36.9)	157 (35.6)	
	25.0–29.9	253 (33.7)	206 (40.6)	173 (39.2)	
	≥ 30.0	193 (25.7)	101 (19.9)	103 (23.4)	
Tumor Histology	Adenocarcinoma	595 (79.3)	430 (84.8)	382 (86.6)	0.002
	Squamous Cell Carcinoma	155 (20.7)	77 (15.2)	59 (13.4)	
Tumor location	Proximal/Middle	61 (8.1)	32 (6.3)	25 (5.7)	0.005
	Distal	238 (31.7)	138 (27.2)	168 (38.1)	
	Siewert 1	239 (31.9)	197 (38.9)	133 (30.2)	
	Siewert 2–3	211 (28.1)	140 (27.6)	115 (26.1)	
	Missing	1 (0.1)	0 (0.0)	0 (0.0)	
Clinical AJCC T Stage	cT1	81 (10.8)	57 (11.2)	50 (11.3)	0.411
	cT2	153 (20.4)	99 (19.5)	107 (24.3)	
	cT3/T4a	516 (68.8)	351 (69.2)	284 (64.4)	
Clinical AJCC N Stage	cN0	313 (41.7)	218 (43.0)	208 (47.2)	0.355
	cN1	295 (39.3)	189 (37.3)	162 (36.7)	
	cN2/3	142 (18.9)	100 (19.7)	71 (16.1)	
Preoperative nutrition	No	411 (54.8)	255 (50.3)	214 (48.5)	0.080
	Yes	339 (45.2)	252 (49.7)	227 (51.5)

**Table 2:** Operative-level clinicopathologic characteristics of patients with esophageal cancers receiving open, hybrid and totally minimally invasive esophagectomy

		**Open,** ***n* = 744**	**Hybrid,** ***n* = 500**	**Totally minimally invasive,** ***n* = 540**	***p*-value**
Operative-level factors					
Neoadjuvant Therapy	None	171 (22.8)	129 (25.4)	119 (27.0)	< 0.001
	Chemoradiotherapy	201 (26.8)	114 (22.5)	184 (41.7)	
	Chemotherapy	378 (50.4)	264 (52.1)	138 (31.3)	
Anastomosis technique	Handsewn	123 (16.4)	38 (7.5)	75 (17.0)	< 0.001
	Linear Stapled	111 (14.8)	99 (19.5)	122 (27.7)	
	Circular stapled	516 (68.8)	370 (73.0)	244 (55.3)	
Gastric tube	Thin (< 5 cm)	327 (43.6)	324 (63.9)	337 (76.4)	< 0.001
	Wide (> 5 cm)	408 (54.4)	174 (34.3)	102 (23.1)	
	Whole Stomach	15 (2.0)	9 (1.8)	2 (0.5)	
Pyloric procedures	Not Performed	369 (49.2)	383 (75.5)	341 (77.3)	< 0.001
	Botox	9 (1.2)	2 (0.4)	28 (6.3)	
	Dilatation	56 (7.5)	63 (12.4)	33 (7.5)	
	Pyloromyotomy	29 (3.9)	9 (1.8)	5 (1.1)	
	Pyloroplasty	287 (38.3)	50 (9.9)	34 (7.7)	
Omentoplasty	No	509 (67.9)	295 (58.2)	191 (43.3)	< 0.001
	Yes	241 (32.1)	212 (41.8)	250 (56.7)	

In the TMIE cohort, ASA grade III-IV patients (40% vs 30% vs 29%, *p* < 0.001), adenocarcinoma histology (87% vs 85% vs 79%, *p* = 0.002), and distal cancers (38% vs 27% v2 32%, *p* = 0.005) were significantly more common than in the HE and OE cohort (Table 1). Also, more TMIE patients, compared with HE and OE, had neoadjuvant chemoradiotherapy (42% vs 23% vs 27%, *p* < 0.001), a linear stapled anastomosis (28% vs 20% vs 15%, *p* < 0.001), thin gastric tube (< 5 cm width) (76% vs 64% vs 44%, *p* < 0.001), and omentoplasty (57% vs 42% vs 32%, *p* < 0.001) (Table 2). There were no significant differences in the rates of clinical AJCC T3/T4a disease (64% vs 69% vs 69%, *p* = 0.4) and clinical AJCC N2/N3 (16% vs 20% vs 19%, *p* = 0.4) disease across patients receiving TMIE, HE and OE.

#### Primary outcome

##### Postoperative pulmonary complications (PPC)

**Fig. 2:**
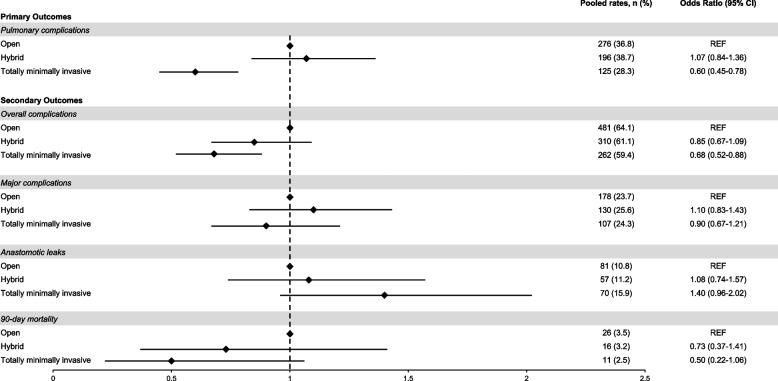
Summary Forest plot of primary (i.e. pulmonary complications) and secondary (i.e. overall & major complications, anastomotic leaks, 90-day mortality) outcomes surgical approaches in patients with transthoracic esophageal cancers

Patients receiving TMIE had significantly lower rates of PPC patients having HE and OE (28% vs 39% vs 37%, *p* = 0.002). On adjusted analyses, patients receiving TMIE had significantly lower rates of PPC than open (OR: 0.60, CI_95%_: 0.45—0.78, *p* < 0.001) (Table 3, Fig. 2). The full model is presented in Supplementary Table 1 (c-statistic 0.71). Sensitivity analyses demonstrated significantly lower rates of PPC with TMIE compared to HE (OR: 0.53, CI_95%_: 0.39—0.73, *p* < 0.001) (Supplementary Table 2). Exploratory analyses identified TMIE patients with PPC were more likely to have higher ASA grade, respiratory disease, distal esophageal cancers, and advanced disease (Supplementary Table 3).

**Table 3:** Univariable and multivariable analysis of postoperative (i.e., pulmonary complications, anastomotic leaks, overall & major complications, and 90-day mortality) outcomes comparing open, hybrid and totally minimally invasive esophagectomy in patients with esophageal cancer

	**Rates, n (%)**	**Univariable, OR (95% CI)**	**Multivariable, OR (95% CI)**
Pulmonary complications^a^			
Open	276 (36.8)	REF	REF
Hybrid	196 (38.7)	1.08 (0.86–1.37, *p* = 0.504)	1.07 (0.84–1.36, *p* = 0.6)
Totally minimally invasive	125 (28.3)	0.68 (0.53–0.88, *p* = 0.003)	0.60 (0.45–0.78, *p* < 0.001)
Overall complications^a^			
Open	481 (64.1)	REF	REF
Hybrid	310 (61.1)	0.88 (0.70–1.11, *p* = 0.282)	0.85 (0.67–1.09, *p* = 0.204)
Totally minimally invasive	262 (59.4)	0.82 (0.64–1.04, *p* = 0.104)	0.68 (0.52–0.88, *p* = 0.004)
Major complications^a^			
Open	178 (23.7)	REF	REF
Hybrid	130 (25.6)	1.11 (0.85–1.44, *p* = 0.441)	1.10 (0.83–1.43, *p* = 0.510)
Totally minimally invasive	107 (24.3)	1.03 (0.78–1.35, *p* = 0.836)	0.90 (0.67–1.21, *p* = 0.489)
Anastomotic leaks^a^			
Open	81 (10.8)	REF	REF
Hybrid	57 (11.2)	1.05 (0.73–1.50, *p* = 0.806)	1.08 (0.74–1.57, *p* = 0.682)
Totally minimally invasive	70 (15.9)	1.56 (1.10–2.20, *p* = 0.012)	1.40 (0.96–2.02, *p* = 0.076)
90-day mortality^a^			
Open	26 (3.5)	REF	REF
Hybrid	16 (3.2)	0.91 (0.47–1.69, *p* = 0.764)	0.73 (0.37–1.41, *p* = 0.352)
Totally minimally invasive	11 (2.5)	0.71 (0.33–1.42, *p* = 0.352)	0.50 (0.22–1.06, *p* = 0.079)

^a^Adjusted for center volume, country income, age at surgery, sex, ASA grade, smoking status, respiratory disease, body mass index, tumor histology & location, clinical AJCC T stage, clinical AJCC N stage, preoperative nutrition, neoadjuvant therapy, anastomotic technique, and surgical approach

#### Secondary outcomes

##### Overall complications

There was no significant difference in rates of overall complications between OE, HE and TMIE (64% vs 61% vs 61%, *p* = 0.2). However, patients receiving TMIE had significantly lower rates of overall complications than OE (OR: 0.68, CI_95%_: 0.52—0.88, *p* = 0.004) on adjusted analyses (Table 3, Fig. 2). The full model is presented in Supplementary Table 4 (c-statistic 0.69). Sensitivity analyses demonstrated no significant difference in overall complications between HE and TMIE in adjusted analyses. (Supplementary Table 1).

##### Major complications

There was no significant difference in rates of major complications between OE, HE and TMIE (24% vs 26% vs 24%, *p* = 0.5). On adjusted analyses, there were no significant difference in rates of major complications between patients receiving TMIE and OE (Table 3, Fig. 2). The full model is presented in Supplementary Table 5 (c-statistic 0.70). Sensitivity analyses demonstrated no significant difference in overall complications between HE and TMIE in adjusted analyses (Supplementary Table 1).

##### Anastomotic leaks

Patients receiving TMIE had significantly higher rates of anastomotic leaks compared to patients having OE and HE (16% vs 11% vs 11%, *p* = 0.026) (Table 3). On adjusted analyses, there were no significant difference in rates of anastomotic leaks in patients receiving HE (OR: 1.08, CI_95%_: 0.74—1.57, *p* = 0.7) and TMIE (OR: 1.40, CI_95%_: 0.96—2.02, *p* = 0.1) compared to OE (Table 3, Fig. 2). The full model is presented in Supplementary Table 6 (c-statistic 0.66). Sensitivity analyses between HE and TMIE demonstrated no significant difference in anastomotic leak rates between the two techniques in adjusted analyses (Supplementary Table 1). Anastomotic leaks were further classified using ECCG definitions, with no significant difference in severity grading across surgical approaches.

##### 90-day mortality

There was no significant difference in rates of 90-day mortality between OE, HE and TMIE (4% vs 3% vs 3%, *p* = 0.6) (Table 3, Fig. 2). On adjusted analyses, there were no significant difference in rates of 90-day mortality between patients receiving TMIE and OE (Table 3). The full model is presented in Supplementary Table 7 (c-statistic 0.75). Sensitivity analyses demonstrated no significant difference in 90-day mortality between HE and TMIE in adjusted analyses (Supplementary Table 1).

##### Other complications

A summary of postoperative complications as defined according to ECCG by OE, HE and TMIE are presented in Supplementary Table 8. There were significant differences in operating time between patient receiving OE, HE and TMIE (median: 350 vs 355 vs 367, *p* < 0.001). There were no significant differences in the overall length of stay between patients receiving OE, HE and TMIE (mean: 17.8 vs 16.7 vs 16.5 days, *p* = 0.2).

#### Sensitivity analysis

##### Respiratory disease

Of 1,784 patients receiving either OE, HE or TMIE, 11% (*n* = 204) had underlying respiratory disease. Baseline characteristics for these patients are presented in Supplementary Table 9. Sensitivity analyses performed for PPC, overall and major complications, anastomotic leaks and 90-day mortality (Supplementary Table 10) were in line with the findings of the main analysis. On adjusted analyses, patients receiving TMIE had significantly lower rates of PPC than (OR: 0.53, CI_95%_: 0.39—0.72, *p* < 0.001) (Supplementary Table 10).

##### Neoadjuvant chemoradiotherapy

Sensitivity analyses were performed in patients receiving neoadjuvant chemoradiotherapy (*n* = 499), of which 37% (*n* = 184) received TMIE. Baseline characteristics for these patients are presented in Supplementary Table 11. Sensitivity analyses performed for PPC, overall and major complications, anastomotic leaks and 90-day mortality (Supplementary Table 12) were in line with the findings of the main analysis.

##### High volume centers

Sensitivity analyses were performed in patients receiving treatment in high volume centers (*n* = 527), of which 30% (*n* = 156) received TMIE. Baseline characteristics for these patients are presented in Supplementary Table 13. Sensitivity analyses performed for PPC, overall complications, anastomotic leaks and 90-day mortality (Supplementary Table 14) were in line with the findings of the main analysis. However, patients receiving TMIE had significantly lower rates of major complications (17% vs 26%, *p* = 0.001) than OE, which remained on adjusted analyses (OR: 0.48, CI_95%_: 0.24—0.94, *p* = 0.037).

## Discussion

Current guidelines from the European Society of Medical Oncology (ESMO) [30] and the National Comprehensive Cancer Network (NCCN) [31] recommend hybrid esophagectomies. However, evidence demonstrating any superiority or safety for TMIE over OE or HE or any other existing surgical approach is lacking. This international cohort study on patients with esophageal cancers demonstrated that TMIE was associated with lower 90-day PPC. There was however no difference in overall complications and anastomotic leak rates in the adjusted analyses. Margin-negative resection rates were significantly higher after TMIE compared to OE or HE, albeit lower than in RCTs [10, 11]. These warrant a further review into causes and mechanisms in selected patients, and that quality assurance in delivery of TMIE.

To date, evidence surrounding impact of MIE on postoperative outcomes, especially PPC compared to OE or HE remains unclear. Evidence from previously published RCT’s comparing OE with HE (i.e. MIRO [11]) or TMIE (i.e. TIME [10], ROBOT [32]) demonstrated significantly lower rates of PPC with HE and TMIE, respectively. However, a published meta-analysis [33] of non-randomized studies comparing HE and TMIE demonstrated no significant difference in rates of pneumonia between the two techniques. A recently published review also emphasises the uncertainty that exists in the current evidence on this topic [34]. These findings were aligned with a recent population-based cohort study from Sweden [13].

Reducing rates of anastomotic leaks remain a major topic of discussion amongst esophageal surgeons and efforts to reduce them remain a priority. However, adoption of TMIE has been associated with significantly higher rates of anastomotic leaks compared to OE. Firstly, a recently published cohort study from the ESOData demonstrated higher anastomotic leaks rate with TMIE than HE [35]. However, the ESOData included patients from high volume, high income countries compared to the present study. Therefore, the present study allowed the true impact of adoption of TMIE from global real-world data. Secondly, a meta-analysis [33] that compared TMIE with HE reported significantly higher rates of anastomotic leaks with transthoracic TMIE compared with HE. In addition, these anastomotic leaks following esophagectomy is linked to reduced long-term overall survival from a recent meta-analysis [7, 8]. However, in the present study, there was a trend towards higher anastomotic leak rates with TMIE compared to OE and HE, as high as 40%. Although the relative risks are higher, the absolute rates of anastomotic leak rates are lower than previously published randomised and non-randomised trials.

Several reasons may explain, the numerically higher rate of anastomotic leaks observed with TMIE, although not statistically significant in adjusted models. Firstly, TMIE involves a technically complex, intracorporeal anastomosis that can be affected by reduced tactile feedback and visualization limitations. Moreover, conduit perfusion can be more difficult to assess accurately in minimally invasive approaches, especially without adjuncts such as ICG fluorescence angiography. Second, stapling techniques and conduit preparation also vary—thin gastric tubes, more commonly used in TMIE, may be more vulnerable to ischemia. Third, learning curve effects cannot be excluded, particularly as TMIE adoption is increasing globally and standardisation of technique remains heterogeneous. Our analysis did not directly measure surgeon experience, but future studies should address this as a modifiable factor.

The present study has some limitations. Firstly, the present study does not capture proficiency gains or learning curves of individual surgeons performing TMIE to assess impact of this on complications. Although interhospital variation has been included into the adjusted model, unknown confounders such as these may also affect postoperative complications. Secondly, impact of surgical approaches on long-term survival data and health-related quality of life were not available in this cohort study. Important endpoints such as dysphagia, reflux, nutritional status, and patient-reported quality of life were not collected. These outcomes are essential to understanding the true burden of surgery from the patient’s perspective, particularly when comparing different minimally invasive approaches. Future collaborative efforts should prioritize the inclusion of longitudinal follow-up with validated quality of life instruments to better inform surgical decision-making. Thirdly, there are potentially unmeasured confounders that were not included in the adjusted models. These include surgeon experience (e.g., learning curves) and hospital-level services such as prehabilitation and enhanced recovery after surgery protocols which may exists in some centers. Variation in some of these measures may affect outcomes in patients undergoing esophagectomy around the world.

## Conclusion

This study provides real-world data that TMIE was associated with lower 90-day PPC than OE and HE approaches, especially in patients with underlying respiratory disease or receiving neoadjuvant chemoradiotherapy. These warrant a further review into causes and mechanisms in selected patients, and that quality assurance in delivery of TMIE is probably of major importance. The ideal surgical approach remains unclear, and ongoing trials will provide more evidence within a few years that may clarify the optimum approach to locally advanced esophageal cancers.

## Supplementary Information

Supplementary Material 1.

## Data Availability

Available on request from corresponding authors.
