# Supplementary material for: Postoperative outcomes after minimally invasive esophagectomy: an international cohort study from the Oesophagogastric Anastomosis Audit (OGAA)
Source: BMC Surg. 2025 May 22;25:225. doi: 10.1186/s12893-025-02941-6 (PMC12096545; doi:10.1186/s12893-025-02941-6)
Supplement: Supplementary file 1 — Supplementary Material 1. [file 12893_2025_2941_MOESM1_ESM.docx]

# Supplementary Table 1 Univariable and multivariable logistic regression for postoperative pulmonary complications in patients receiving open, hybrid and totally minimally invasive transthoracic esophagectomy for esophageal cancers

|  |  | **OR (univariable)** | **OR (multivariable)** |
| --- | --- | --- | --- |
| **Hospital-level factors** |  |  |  |
| Center volume | <28 | - | - |
|  | 28-50 | 1.27 (1.00-1.61, p=0.055) | 1.26 (0.97-1.64, p=0.081) |
|  | ≥51 | 0.96 (0.74-1.24, p=0.761) | 0.92 (0.70-1.22, p=0.575) |
| Country income | High income country | - | - |
|  | Low-Middle income country | 0.60 (0.35-0.99, p=0.054) | 0.85 (0.46-1.53, p=0.601) |
| **Patient-level factors** |  |  |  |
| Age at surgery |  | 1.02 (1.01-1.03, p=0.002) | 1.01 (1.00-1.02, p=0.034) |
| Sex | Female | - | - |
|  | Male | 1.23 (0.95-1.60, p=0.124) | 1.05 (0.79-1.39, p=0.761) |
| ASA Grade | 1 | - | - |
|  | 2 | 1.44 (1.04-2.02, p=0.031) | 1.27 (0.90-1.81, p=0.183) |
|  | 3-4 | 2.11 (1.50-3.00, p<0.001) | 1.81 (1.24-2.65, p=0.002) |
| Smoking status | Never smoker | - | - |
|  | Ex-smoker | 1.36 (1.09-1.71, p=0.007) | 1.28 (1.01-1.63, p=0.039) |
|  | Current smoker | 1.32 (0.97-1.80, p=0.071) | 1.35 (0.97-1.89, p=0.073) |
|  | Unknown | 1.42 (0.77-2.57, p=0.250) | 1.19 (0.63-2.19, p=0.590) |
| Respiratory Disease | No | - | - |
|  | Yes | 1.76 (1.31-2.37, p<0.001) | 1.50 (1.10-2.06, p=0.011) |
| Body mass index | ≤18.5 | - | - |
|  | 18.6-24.9 | 1.85 (0.96-3.85, p=0.079) | 1.92 (0.98-4.08, p=0.071) |
|  | 25.0-29.9 | 2.07 (1.08-4.30, p=0.037) | 2.30 (1.15-4.93, p=0.024) |
|  | ≥30.0 | 2.66 (1.37-5.57, p=0.006) | 2.86 (1.42-6.22, p=0.005) |
| Tumor Histology | Adenocarcinoma | - | - |
|  | Squamous Cell Carcinoma | 0.86 (0.65-1.12, p=0.263) | 1.00 (0.70-1.41, p=0.984) |
| Tumor location | Proximal/Middle | - | - |
|  | Distal | 1.10 (0.73-1.68, p=0.652) | 1.02 (0.64-1.63, p=0.949) |
|  | Siewert 1 | 1.03 (0.68-1.57, p=0.905) | 0.92 (0.56-1.52, p=0.746) |
|  | Siewert 2-3 | 0.93 (0.61-1.43, p=0.727) | 0.85 (0.51-1.42, p=0.534) |
| Clinical AJCC T Stage | cT1 | - | - |
|  | cT2 | 1.30 (0.90-1.88, p=0.166) | 1.43 (0.96-2.15, p=0.080) |
|  | cT3/T4a | 1.02 (0.74-1.43, p=0.883) | 1.12 (0.75-1.68, p=0.574) |
| Clinical AJCC N Stage | cN0 | - | - |
|  | cN1 | 0.89 (0.71-1.11, p=0.286) | 0.83 (0.65-1.06, p=0.139) |
|  | cN2/3 | 0.87 (0.65-1.14, p=0.314) | 0.85 (0.62-1.15, p=0.289) |
| Preoperative nutrition | No | - | - |
|  | Yes | 1.08 (0.88-1.32, p=0.452) | 1.18 (0.95-1.47, p=0.127) |
| **Operative Factors** |  |  |  |
| Neoadjuvant Therapy | None | - | - |
|  | Chemoradiotherapy | 1.02 (0.77-1.34, p=0.914) | 1.12 (0.80-1.58, p=0.514) |
|  | Chemotherapy | 1.09 (0.85-1.40, p=0.485) | 1.17 (0.85-1.62, p=0.338) |
| Anastomotic Technique | Handsewn | - | - |
|  | Linear Stapled | 0.97 (0.68-1.37, p=0.849) | 0.86 (0.59-1.24, p=0.414) |
|  | Circular stapled | 0.95 (0.71-1.28, p=0.756) | 0.78 (0.57-1.07, p=0.117) |
| Surgical approach | Open | - | - |
|  | Hybrid | 1.08 (0.86-1.37, p=0.504) | 1.07 (0.84-1.36, p=0.589) |
|  | Totally minimally invasive | 0.68 (0.53-0.88, p=0.003) | 0.60 (0.45-0.78, p<0.001) |

# Supplementary Table 2 Univariable and multivariable analysis of postoperative (i.e. pulmonary complications, anastomotic leaks, overall & major complications, and 90-day mortality) outcomes comparing hybrid and totally minimally invasive esophagectomy in patients with esophageal cancer as sensitivity analyses

|  | **Rates, n (%)** | **Univariable, OR (95% CI)** | **Multivariable, OR (95% CI)** |
| --- | --- | --- | --- |
| **Pulmonary complications*** |  |  |  |
| Hybrid | 196 (38.7) | REF | REF |
| Totally minimally invasive | 125 (28.3) | 0.63 (0.48-0.82, p=0.001) | 0.53 (0.39-0.73, p<0.001) |
|  |  |  |  |
| **Overall complications*** |  |  |  |
| Hybrid | 310 (61.1) | REF | REF |
| Totally minimally invasive | 262 (59.4) | 0.93 (0.72-1.21, p=0.586) | 0.76 (0.56-1.03, p=0.073) |
|  |  |  |  |
| **Major complications*** |  |  |  |
| Hybrid | 52 (10.3) | REF | REF |
| Totally minimally invasive | 43 (9.8) | 0.95 (0.62-1.45, p=0.796) | 0.85 (0.53-1.37, p=0.513) |
|  |  |  |  |
| **Anastomotic leaks*** |  |  |  |
| Hybrid | 57 (11.2) | REF | REF |
| Totally minimally invasive | 70 (15.9) | 1.49 (1.02-2.17, p=0.038) | 1.18 (0.78-1.79, p=0.431) |
|  |  |  |  |
| **90-day mortality*** |  |  |  |
| Hybrid | 16 (3.2) | REF | REF |
| Totally minimally invasive | 11 (2.5) | 0.79 (0.35-1.70, p=0.542) | 0.73 (0.29-1.77, p=0.492) |

**Adjusted for center volume, country income, age at surgery, sex, ASA grade, smoking status, respiratory disease, body mass index, tumor histology & location, clinical AJCC T stage, clinical AJCC N stage, preoperative nutrition, neoadjuvant therapy, anastomotic technique, and surgical approach*

# Supplementary Table 3 Summary of hospital-, patient-, and operative-level clinicopathologic characteristics in patients undergoing esophagectomy for esophageal cancers by surgical approach

|  |  | **Open** | | | **Hybrid** | | | **Totally minimally invasive** | | |
| --- | --- | --- | --- | --- | --- | --- | --- | --- | --- | --- |
|  |  | No | Yes | p-value | No | Yes | p-value | No | Yes | p-value |
| **Hospital-level factors** |  |  |  |  |  |  |  |  |  |  |
| Center volume | <28 | 151 (31.9) | 80 (29.0) | 0.3 | 84 (27.0) | 45 (23.0) | 0.6 | 108 (34.2) | 47 (37.6) | 0.2 |
|  | 28-50 | 187 (39.5) | 126 (45.7) |  | 126 (40.5) | 86 (43.9) |  | 88 (27.8) | 42 (33.6) |  |
|  | ≥51 | 135 (28.5) | 70 (25.4) |  | 101 (32.5) | 65 (33.2) |  | 120 (38.0) | 36 (28.8) |  |
| Country income | High income country | 430 (90.9) | 265 (96.0) | **0.014** | 306 (98.4) | 192 (98.0) | 1.0 | 304 (96.2) | 120 (96.0) | 1.0 |
|  | Low-Middle income country | 43 (9.1) | 11 (4.0) |  | 5 (1.6) | 4 (2.0) |  | 12 (3.8) | 5 (4.0) |  |
| **Patient-level factors** |  |  |  |  |  |  |  |  |  |  |
| Age at surgery |  | 63.1 (10.6) | 65.1 (8.9) | **0.011** | 65.6 (9.8) | 66.7 (9.3) | 0.2 | 64.3 (10.0) | 65.7 (9.3) | 0.2 |
| Sex | Female | 103 (21.8) | 51 (18.5) | 0.3 | 57 (18.3) | 32 (16.3) | 0.6 | 57 (18.0) | 17 (13.6) | 0.3 |
|  | Male | 370 (78.2) | 225 (81.5) |  | 254 (81.7) | 164 (83.7) |  | 259 (82.0) | 108 (86.4) |  |
| ASA Grade | 1 | 86 (18.2) | 22 (8.0) | **<0.001** | 48 (15.4) | 24 (12.2) | 0.4 | 28 (8.9) | 10 (8.0) | **0.003** |
|  | 2 | 269 (56.9) | 153 (55.4) |  | 176 (56.6) | 108 (55.1) |  | 176 (55.7) | 49 (39.2) |  |
|  | 3-4 | 118 (24.9) | 101 (36.6) |  | 87 (28.0) | 64 (32.7) |  | 112 (35.4) | 66 (52.8) |  |
| Smoking status | Never smoker | 208 (44.0) | 91 (33.0) | **0.019** | 119 (38.3) | 62 (31.6) | 0.4 | 103 (32.6) | 39 (31.2) | 0.4 |
|  | Ex-smoker | 188 (39.7) | 132 (47.8) |  | 140 (45.0) | 94 (48.0) |  | 151 (47.8) | 65 (52.0) |  |
|  | Current smoker | 57 (12.1) | 43 (15.6) |  | 45 (14.5) | 34 (17.3) |  | 59 (18.7) | 18 (14.4) |  |
|  | Unknown | 20 (4.2) | 10 (3.6) |  | 7 (2.3) | 6 (3.1) |  | 3 (0.9) | 3 (2.4) |  |
| Respiratory Disease | No | 427 (90.3) | 232 (84.1) | **0.016** | 283 (91.0) | 174 (88.8) | 0.5 | 282 (89.2) | 95 (76.0) | **0.001** |
|  | Yes | 46 (9.7) | 44 (15.9) |  | 28 (9.0) | 22 (11.2) |  | 34 (10.8) | 30 (24.0) |  |
| Body Mass Index, kg/m^2^ | ≤18.5 | 26 (5.5) | 6 (2.2) | **0.005** | 9 (2.9) | 4 (2.0) | 0.9 | 7 (2.2) | 1 (0.8) | 0.1 |
|  | 18.6-24.9 | 185 (39.1) | 86 (31.2) |  | 112 (36.0) | 75 (38.3) |  | 117 (37.0) | 40 (32.0) |  |
|  | 25.0-29.9 | 155 (32.8) | 98 (35.5) |  | 127 (40.8) | 79 (40.3) |  | 128 (40.5) | 45 (36.0) |  |
|  | ≥30.0 | 107 (22.6) | 86 (31.2) |  | 63 (20.3) | 38 (19.4) |  | 64 (20.3) | 39 (31.2) |  |
| Tumor Histology | Adenocarcinoma | 364 (77.0) | 231 (83.7) | **0.031** | 261 (83.9) | 169 (86.2) | 0.6 | 279 (88.3) | 103 (82.4) | 0.1 |
|  | Squamous Cell Carcinoma | 109 (23.0) | 45 (16.3) |  | 50 (16.1) | 27 (13.8) |  | 37 (11.7) | 22 (17.6) |  |
| Tumor location | Proximal/Middle | 42 (8.9) | 19 (6.9) | 0.8 | 20 (6.4) | 12 (6.1) | 1.0 | 15 (4.7) | 10 (8.0) | **0.027** |
|  | Distal | 148 (31.3) | 90 (32.6) |  | 84 (27.0) | 54 (27.6) |  | 111 (35.1) | 57 (45.6) |  |
|  | Siewert 1 | 150 (31.7) | 89 (32.2) |  | 121 (38.9) | 76 (38.8) |  | 97 (30.7) | 36 (28.8) |  |
|  | Siewert 2-3 | 133 (28.1) | 78 (28.3) |  | 86 (27.7) | 54 (27.6) |  | 93 (29.4) | 22 (17.6) |  |
| Clinical AJCC T Stage | cT1 | 50 (10.6) | 31 (11.2) | 1.0 | 38 (12.2) | 19 (9.7) | 0.5 | 37 (11.7) | 13 (10.4) | **0.003** |
|  | cT2 | 97 (20.5) | 56 (20.3) |  | 57 (18.3) | 42 (21.4) |  | 63 (19.9) | 44 (35.2) |  |
|  | cT3/T4a | 326 (68.9) | 189 (68.5) |  | 216 (69.5) | 135 (68.9) |  | 216 (68.4) | 68 (54.4) |  |
| Clinical AJCC N Stage | cN0 | 185 (39.1) | 127 (46.0) | 0.2 | 135 (43.4) | 83 (42.3) | 1.0 | 146 (46.2) | 62 (49.6) | 0.5 |
|  | cN1 | 196 (41.4) | 99 (35.9) |  | 115 (37.0) | 74 (37.8) |  | 115 (36.4) | 47 (37.6) |  |
|  | cN2/3 | 92 (19.5) | 50 (18.1) |  | 61 (19.6) | 39 (19.9) |  | 55 (17.4) | 16 (12.8) |  |
| Preoperative nutrition | No | 268 (56.7) | 143 (51.8) | 0.2 | 154 (49.5) | 101 (51.5) | 0.7 | 156 (49.4) | 58 (46.4) | 0.6 |
|  | Yes | 205 (43.3) | 133 (48.2) |  | 157 (50.5) | 95 (48.5) |  | 160 (50.6) | 67 (53.6) |  |
| **Operative-level factors** |  |  |  |  |  |  |  |  |  |  |
| Neoadjuvant Therapy | None | 107 (22.6) | 64 (23.2) | 1.0 | 83 (26.7) | 46 (23.5) | 0.6 | 86 (27.2) | 33 (26.4) | 0.9 |
|  | Chemoradiotherapy | 126 (26.6) | 75 (27.2) |  | 71 (22.8) | 43 (21.9) |  | 130 (41.1) | 54 (43.2) |  |
|  | Chemotherapy | 240 (50.7) | 137 (49.6) |  | 157 (50.5) | 107 (54.6) |  | 100 (31.6) | 38 (30.4) |  |
| Anastomosis technique | Handsewn | 80 (16.9) | 43 (15.6) | 0.5 | 15 (4.8) | 23 (11.7) | **0.015** | 56 (17.7) | 19 (15.2) | **0.026** |
|  | Linear Stapled | 75 (15.9) | 36 (13.0) |  | 64 (20.6) | 35 (17.9) |  | 76 (24.1) | 46 (36.8) |  |
|  | Circular stapled | 318 (67.2) | 197 (71.4) |  | 232 (74.6) | 138 (70.4) |  | 184 (58.2) | 60 (48.0) |  |
| Gastric tube | Thin (<5cm) | 204 (43.1) | 122 (44.2) | 0.4 | 208 (66.9) | 116 (59.2) | 0.2 | 247 (78.2) | 90 (72.0) | 0.2 |
|  | Wide (>5cm) | 257 (54.3) | 151 (54.7) |  | 97 (31.2) | 77 (39.3) |  | 67 (21.2) | 35 (28.0) |  |
|  | Whole Stomach | 12 (2.5) | 3 (1.1) |  | 6 (1.9) | 3 (1.5) |  | 2 (0.6) | 0 (0.0) |  |
| Pyloric procedures | Not Performed | 227 (48.0) | 141 (51.1) | 0.8 | 228 (73.3) | 155 (79.1) | **0.027** | 249 (78.8) | 92 (73.6) | 0.3 |
|  | Botox | 5 (1.1) | 4 (1.4) |  | 0 (0.0) | 2 (1.0) |  | 22 (7.0) | 6 (4.8) |  |
|  | Dilatation | 36 (7.6) | 20 (7.2) |  | 45 (14.5) | 18 (9.2) |  | 21 (6.6) | 12 (9.6) |  |
|  | Pyloromyotomy | 21 (4.4) | 8 (2.9) |  | 3 (1.0) | 6 (3.1) |  | 2 (0.6) | 3 (2.4) |  |
|  | Pyloroplasty | 184 (38.9) | 103 (37.3) |  | 35 (11.3) | 15 (7.7) |  | 22 (7.0) | 12 (9.6) |  |
| Omentoplasty | No | 328 (69.3) | 180 (65.2) | 0.3 | 175 (56.3) | 120 (61.2) | 0.3 | 135 (42.7) | 56 (44.8) | 0.8 |
|  | Yes | 145 (30.7) | 96 (34.8) |  | 136 (43.7) | 76 (38.8) |  | 181 (57.3) | 69 (55.2) |  |

*Abbreviations: ARDS: acute respiratory distress syndrome, SSI: surgical site infection*

# Supplementary Table 4 Univariable and multivariable logistic regression for overall complications in patients receiving open, hybrid and totally minimally invasive transthoracic esophagectomy for esophageal cancers

|  |  | **OR (univariable)** | **OR (multivariable)** |
| --- | --- | --- | --- |
| **Hospital-level factors** |  |  |  |
| Center volume | <28 | - | - |
|  | 28-50 | 1.49 (1.17-1.89, p=0.001) | 1.45 (1.12-1.88, p=0.005) |
|  | ≥51 | 1.31 (1.02-1.67, p=0.035) | 1.30 (0.99-1.70, p=0.059) |
| Country income | High income country | - | - |
|  | Low-Middle income country | 0.46 (0.29-0.72, p=0.001) | 0.60 (0.35-1.04, p=0.069) |
| **Patient-level factors** |  |  |  |
| Age at surgery |  | 1.01 (1.00-1.02, p=0.018) | 1.01 (1.00-1.02, p=0.134) |
| Sex | Female | - | - |
|  | Male | 0.97 (0.75-1.25, p=0.818) | 0.87 (0.65-1.15, p=0.322) |
| ASA Grade | 1 | - | - |
|  | 2 | 1.20 (0.89-1.62, p=0.224) | 1.00 (0.73-1.38, p=0.983) |
|  | 3-4 | 1.82 (1.31-2.51, p<0.001) | 1.51 (1.05-2.16, p=0.026) |
| Smoking status | Never smoker | - | - |
|  | Ex-smoker | 1.42 (1.15-1.77, p=0.001) | 1.38 (1.09-1.74, p=0.007) |
|  | Current smoker | 1.39 (1.03-1.88, p=0.033) | 1.45 (1.05-2.02, p=0.026) |
|  | Unknown | 1.73 (0.94-3.34, p=0.086) | 1.66 (0.88-3.27, p=0.125) |
| Respiratory Disease | No | - | - |
|  | Yes | 1.92 (1.39-2.70, p<0.001) | 1.73 (1.23-2.48, p=0.002) |
| Body mass index | ≤18.5 | - | - |
|  | 18.6-24.9 | 1.67 (0.95-2.95, p=0.073) | 1.87 (1.02-3.44, p=0.042) |
|  | 25.0-29.9 | 1.76 (1.00-3.10, p=0.049) | 2.12 (1.14-3.96, p=0.017) |
|  | ≥30.0 | 2.47 (1.38-4.43, p=0.002) | 3.01 (1.59-5.72, p=0.001) |
| Tumor Histology | Adenocarcinoma | - | - |
|  | Squamous Cell Carcinoma | 1.06 (0.82-1.39, p=0.639) | 1.13 (0.80-1.61, p=0.474) |
| Tumor location | Proximal/Middle | - | - |
|  | Distal | 0.83 (0.54-1.26, p=0.394) | 0.76 (0.47-1.21, p=0.255) |
|  | Siewert 1 | 0.64 (0.42-0.98, p=0.042) | 0.62 (0.38-1.02, p=0.065) |
|  | Siewert 2-3 | 0.74 (0.48-1.13, p=0.170) | 0.74 (0.44-1.23, p=0.251) |
| Clinical AJCC T Stage | cT1 | - | - |
|  | cT2 | 1.18 (0.83-1.69, p=0.355) | 1.21 (0.81-1.79, p=0.349) |
|  | cT3/T4a | 1.30 (0.95-1.77, p=0.102) | 1.31 (0.89-1.93, p=0.176) |
| Clinical AJCC N Stage | cN0 | - | - |
|  | cN1 | 1.06 (0.85-1.32, p=0.599) | 0.96 (0.75-1.22, p=0.714) |
|  | cN2/3 | 1.08 (0.82-1.42, p=0.588) | 1.05 (0.77-1.42, p=0.767) |
| Preoperative nutrition | No | - | - |
|  | Yes | 1.04 (0.85-1.26, p=0.709) | 1.07 (0.86-1.32, p=0.546) |
| **Operative Factors** |  |  |  |
| Neoadjuvant Therapy | None | - | - |
|  | Chemoradiotherapy | 1.37 (1.05-1.79, p=0.022) | 1.26 (0.90-1.76, p=0.185) |
|  | Chemotherapy | 1.21 (0.95-1.54, p=0.124) | 1.11 (0.81-1.53, p=0.502) |
| Anastomotic Technique | Handsewn | - | - |
|  | Linear Stapled | 1.45 (1.02-2.06, p=0.039) | 1.30 (0.89-1.90, p=0.172) |
|  | Circular stapled | 0.91 (0.68-1.21, p=0.527) | 0.71 (0.52-0.98, p=0.036) |
| Surgical approach | Open | - | - |
|  | Hybrid | 0.88 (0.70-1.11, p=0.282) | 0.85 (0.67-1.09, p=0.204) |
|  | Totally minimally invasive | 0.82 (0.64-1.04, p=0.104) | 0.68 (0.52-0.88, p=0.004) |

# Supplementary Table 5 Univariable and multivariable logistic regression for major complications in patients receiving open, hybrid and totally minimally invasive transthoracic esophagectomy for esophageal cancers

|  |  | **OR (univariable)** | **OR (multivariable)** |
| --- | --- | --- | --- |
| **Hospital-level factors** |  |  |  |
| Center volume | <28 | - | - |
|  | 28-50 | 0.98 (0.70-1.39, p=0.919) | 1.06 (0.73-1.54, p=0.755) |
|  | ≥51 | 0.77 (0.52-1.12, p=0.171) | 0.79 (0.53-1.19, p=0.257) |
| Country income | High income country | - | - |
|  | Low-Middle income country | 1.15 (0.57-2.12, p=0.675) | 0.85 (0.37-1.83, p=0.690) |
| **Patient-level factors** |  |  |  |
| Age at surgery |  | 1.00 (0.99-1.02, p=0.634) | 1.00 (0.98-1.02, p=0.989) |
| Sex | Female | - | - |
|  | Male | 1.49 (1.00-2.29, p=0.060) | 1.46 (0.95-2.32, p=0.096) |
| ASA Grade | 1 | - | - |
|  | 2 | 1.03 (0.65-1.70, p=0.902) | 0.96 (0.59-1.63, p=0.882) |
|  | 3-4 | 1.53 (0.95-2.56, p=0.087) | 1.17 (0.69-2.04, p=0.574) |
| Smoking status | Never smoker | - | - |
|  | Ex-smoker | 1.31 (0.94-1.83, p=0.109) | 1.20 (0.85-1.71, p=0.299) |
|  | Current smoker | 1.45 (0.93-2.22, p=0.092) | 1.28 (0.80-2.04, p=0.296) |
|  | Unknown | 0.76 (0.22-1.96, p=0.615) | 0.75 (0.22-1.97, p=0.596) |
| Respiratory Disease | No | - | - |
|  | Yes | 2.15 (1.47-3.10, p<0.001) | 1.84 (1.22-2.72, p=0.003) |
| Body mass index | ≤18.5 | - | - |
|  | 18.6-24.9 | 1.13 (0.48-3.35, p=0.799) | 1.08 (0.44-3.29, p=0.872) |
|  | 25.0-29.9 | 1.35 (0.57-3.98, p=0.535) | 1.45 (0.57-4.45, p=0.471) |
|  | ≥30.0 | 1.71 (0.71-5.07, p=0.274) | 1.81 (0.70-5.63, p=0.256) |
| Tumor Histology | Adenocarcinoma | - | - |
|  | Squamous Cell Carcinoma | 1.22 (0.83-1.74, p=0.294) | 1.34 (0.82-2.16, p=0.229) |
| Tumor location | Proximal/Middle | - | - |
|  | Distal | 0.83 (0.49-1.48, p=0.514) | 0.80 (0.43-1.52, p=0.481) |
|  | Siewert 1 | 0.67 (0.39-1.20, p=0.160) | 0.70 (0.37-1.39, p=0.299) |
|  | Siewert 2-3 | 0.61 (0.35-1.11, p=0.093) | 0.65 (0.33-1.31, p=0.218) |
| Clinical AJCC T Stage | cT1 | - | - |
|  | cT2 | 1.24 (0.74-2.13, p=0.417) | 1.73 (0.99-3.11, p=0.058) |
|  | cT3/T4a | 0.93 (0.59-1.52, p=0.761) | 1.65 (0.94-2.99, p=0.090) |
| Clinical AJCC N Stage | cN0 | - | - |
|  | cN1 | 0.88 (0.64-1.21, p=0.445) | 0.95 (0.66-1.35, p=0.762) |
|  | cN2/3 | 0.83 (0.55-1.25, p=0.385) | 0.96 (0.60-1.50, p=0.845) |
| Preoperative nutrition | No | - | - |
|  | Yes | 0.85 (0.64-1.14, p=0.286) | 0.97 (0.71-1.32, p=0.832) |
| **Operative Factors** |  |  |  |
| Neoadjuvant Therapy | None | - | - |
|  | Chemoradiotherapy | 0.80 (0.55-1.16, p=0.237) | 0.69 (0.43-1.10, p=0.119) |
|  | Chemotherapy | 0.56 (0.39-0.80, p=0.001) | 0.57 (0.37-0.90, p=0.014) |
| Anastomotic Technique | Handsewn | - | - |
|  | Linear Stapled | 1.04 (0.64-1.71, p=0.885) | 1.00 (0.59-1.69, p=0.988) |
|  | Circular stapled | 0.87 (0.58-1.35, p=0.531) | 0.88 (0.57-1.39, p=0.573) |
| Surgical approach | Open | - | - |
|  | Hybrid | 1.05 (0.73-1.50, p=0.806) | 1.08 (0.74-1.57, p=0.682) |
|  | Totally minimally invasive | 1.56 (1.10-2.20, p=0.012) | 1.40 (0.96-2.02, p=0.076) |

# Supplementary Table 6 Univariable and multivariable logistic regression for anastomotic leaks in patients receiving open, hybrid and totally minimally invasive transthoracic esophagectomy for esophageal cancers

|  |  | **OR (univariable)** | **OR (multivariable)** |
| --- | --- | --- | --- |
| **Hospital-level factors** |  |  |  |
| Center volume | <28 | - | - |
|  | 28-50 | 0.97 (0.74-1.27, p=0.846) | 0.99 (0.74-1.32, p=0.943) |
|  | ≥51 | 1.00 (0.76-1.33, p=0.983) | 1.05 (0.78-1.43, p=0.733) |
| Country income | High income country | - | - |
|  | Low-Middle income country | 0.76 (0.42-1.30, p=0.345) | 1.04 (0.53-1.97, p=0.896) |
| **Patient-level factors** |  |  |  |
| Age at surgery |  | 1.01 (1.00-1.02, p=0.037) | 1.01 (0.99-1.02, p=0.261) |
| Sex | Female | - | - |
|  | Male | 1.11 (0.83-1.48, p=0.495) | 1.08 (0.79-1.49, p=0.626) |
| ASA Grade | 1 | - | - |
|  | 2 | 1.40 (0.96-2.08, p=0.090) | 1.30 (0.87-1.96, p=0.208) |
|  | 3-4 | 2.22 (1.51-3.34, p<0.001) | 1.95 (1.28-3.02, p=0.002) |
| Smoking status | Never smoker | - | - |
|  | Ex-smoker | 1.26 (0.98-1.61, p=0.069) | 1.14 (0.88-1.48, p=0.325) |
|  | Current smoker | 1.20 (0.85-1.67, p=0.301) | 1.10 (0.76-1.58, p=0.602) |
|  | Unknown | 0.79 (0.35-1.60, p=0.538) | 0.73 (0.32-1.51, p=0.426) |
| Respiratory Disease | No | - | - |
|  | Yes | 1.45 (1.05-1.99, p=0.023) | 1.26 (0.89-1.76, p=0.182) |
| Body mass index | ≤18.5 | - | - |
|  | 18.6-24.9 | 1.56 (0.78-3.48, p=0.239) | 1.69 (0.82-3.86, p=0.179) |
|  | 25.0-29.9 | 1.48 (0.74-3.31, p=0.298) | 1.73 (0.82-4.00, p=0.170) |
|  | ≥30.0 | 1.87 (0.92-4.22, p=0.101) | 2.17 (1.02-5.08, p=0.056) |
| Tumor Histology | Adenocarcinoma | - | - |
|  | Squamous Cell Carcinoma | 1.27 (0.95-1.67, p=0.104) | 1.33 (0.91-1.91, p=0.133) |
| Tumor location | Proximal/Middle | - | - |
|  | Distal | 0.88 (0.57-1.37, p=0.561) | 0.95 (0.59-1.56, p=0.838) |
|  | Siewert 1 | 0.69 (0.45-1.08, p=0.097) | 0.78 (0.46-1.32, p=0.346) |
|  | Siewert 2-3 | 0.69 (0.44-1.09, p=0.106) | 0.80 (0.47-1.37, p=0.404) |
| Clinical AJCC T Stage | cT1 | - | - |
|  | cT2 | 0.94 (0.63-1.41, p=0.745) | 1.02 (0.66-1.59, p=0.923) |
|  | cT3/T4a | 0.90 (0.64-1.29, p=0.554) | 1.07 (0.70-1.66, p=0.749) |
| Clinical AJCC N Stage | cN0 | - | - |
|  | cN1 | 0.81 (0.63-1.04, p=0.096) | 0.78 (0.60-1.03, p=0.078) |
|  | cN2/3 | 0.98 (0.72-1.32, p=0.886) | 0.98 (0.70-1.36, p=0.893) |
| Preoperative nutrition | No | - | - |
|  | Yes | 0.94 (0.75-1.17, p=0.578) | 0.95 (0.75-1.21, p=0.677) |
| **Operative Factors** |  |  |  |
| Neoadjuvant Therapy | None | - | - |
|  | Chemoradiotherapy | 1.11 (0.83-1.49, p=0.493) | 1.14 (0.79-1.66, p=0.487) |
|  | Chemotherapy | 0.87 (0.66-1.14, p=0.306) | 1.01 (0.71-1.44, p=0.954) |
| Anastomotic Technique | Handsewn | - | - |
|  | Linear Stapled | 1.50 (1.01-2.25, p=0.049) | 1.46 (0.97-2.24, p=0.075) |
|  | Circular stapled | 1.31 (0.93-1.87, p=0.125) | 1.23 (0.86-1.79, p=0.261) |
| Surgical approach | Open | - | - |
|  | Hybrid | 1.11 (0.85-1.44, p=0.441) | 1.10 (0.83-1.43, p=0.510) |
|  | Totally minimally invasive | 1.03 (0.78-1.35, p=0.836) | 0.90 (0.67-1.21, p=0.489) |

# Supplementary Table 7 Univariable and multivariable logistic regression for 90-day mortality in patients receiving open, hybrid and totally minimally invasive transthoracic esophagectomy for esophageal cancers

|  |  | **OR (univariable)** | **OR (multivariable)** |
| --- | --- | --- | --- |
| **Hospital-level factors** |  |  |  |
| Center volume | <28 | - | - |
|  | 28-50 | 1.02 (0.54-1.97, p=0.952) | 0.98 (0.49-2.01, p=0.962) |
|  | ≥51 | 0.80 (0.38-1.64, p=0.545) | 0.90 (0.41-1.99, p=0.802) |
| Country income | High income country | - | - |
|  | Low-Middle income country | 1.22 (0.29-3.42, p=0.741) | 1.17 (0.22-4.60, p=0.831) |
| **Patient-level factors** |  |  |  |
| Age at surgery |  | 1.06 (1.03-1.10, p<0.001) | 1.06 (1.03-1.11, p=0.001) |
| Sex | Female | - | - |
|  | Male | 0.99 (0.51-2.11, p=0.979) | 1.15 (0.55-2.66, p=0.720) |
| ASA Grade | 1 | - | - |
|  | 2 | 0.55 (0.24-1.34, p=0.158) | 0.42 (0.17-1.10, p=0.063) |
|  | 3-4 | 1.31 (0.61-3.13, p=0.516) | 0.85 (0.35-2.25, p=0.722) |
| Smoking status | Never smoker | - | - |
|  | Ex-smoker | 0.89 (0.48-1.65, p=0.702) | 0.95 (0.49-1.85, p=0.872) |
|  | Current smoker | 1.23 (0.54-2.60, p=0.606) | 1.69 (0.68-4.01, p=0.243) |
|  | Unknown | 0.63 (0.03-3.12, p=0.653) | 0.65 (0.03-3.51, p=0.688) |
| Respiratory Disease | No | - | - |
|  | Yes | 1.12 (0.46-2.36, p=0.786) | 0.96 (0.37-2.17, p=0.928) |
| Body mass index | ≤18.5 | - | - |
|  | 18.6-24.9 | 0.90 (0.25-5.73, p=0.889) | 1.42 (0.34-9.95, p=0.669) |
|  | 25.0-29.9 | 0.79 (0.22-5.05, p=0.756) | 1.36 (0.30-9.93, p=0.721) |
|  | ≥30.0 | 0.73 (0.19-4.78, p=0.683) | 1.40 (0.29-10.71, p=0.704) |
| Tumor Histology | Adenocarcinoma | - | - |
|  | Squamous Cell Carcinoma | 1.43 (0.71-2.68, p=0.282) | 1.07 (0.41-2.58, p=0.880) |
| Tumor location | Proximal/Middle | - | - |
|  | Distal | 0.48 (0.20-1.28, p=0.115) | 0.45 (0.16-1.36, p=0.143) |
|  | Siewert 1 | 0.49 (0.21-1.29, p=0.120) | 0.55 (0.18-1.77, p=0.301) |
|  | Siewert 2-3 | 0.46 (0.18-1.24, p=0.101) | 0.46 (0.15-1.56, p=0.200) |
| Clinical AJCC T Stage | cT1 | - | - |
|  | cT2 | 0.51 (0.17-1.52, p=0.220) | 0.56 (0.18-1.77, p=0.315) |
|  | cT3/T4a | 0.91 (0.42-2.24, p=0.815) | 1.29 (0.49-3.76, p=0.617) |
| Clinical AJCC N Stage | cN0 | - | - |
|  | cN1 | 0.74 (0.38-1.42, p=0.371) | 0.82 (0.39-1.69, p=0.592) |
|  | cN2/3 | 1.57 (0.79-3.02, p=0.185) | 2.16 (1.00-4.64, p=0.048) |
| Preoperative nutrition | No | - | - |
|  | Yes | 0.82 (0.47-1.42, p=0.480) | 0.86 (0.47-1.56, p=0.620) |
| **Operative Factors** |  |  |  |
| Neoadjuvant Therapy | None | - | - |
|  | Chemoradiotherapy | 0.83 (0.43-1.60, p=0.582) | 0.83 (0.35-1.96, p=0.666) |
|  | Chemotherapy | 0.41 (0.20-0.82, p=0.012) | 0.42 (0.17-1.00, p=0.049) |
| Anastomotic Technique | Handsewn | - | - |
|  | Linear Stapled | 2.49 (0.97-7.67, p=0.077) | 3.03 (1.07-10.19, p=0.050) |
|  | Circular stapled | 1.30 (0.55-3.85, p=0.587) | 1.22 (0.48-3.87, p=0.699) |
| Surgical approach | Open | - | - |
|  | Hybrid | 0.91 (0.47-1.69, p=0.764) | 0.73 (0.37-1.41, p=0.352) |
|  | Totally minimally invasive | 0.71 (0.33-1.42, p=0.352) | 0.50 (0.22-1.06, p=0.079) |

# Supplementary Table 8 Summary of postoperative complications according to the Esophagectomy Complications Consensus Group (ECCG) in patients undergoing esophagectomy for esophageal cancers by type of surgical approach

|  | **Open,**  **n=744** | **Hybrid,**  **n=500** | **Totally minimally invasive,**  **n=540** | **p-value** |
| --- | --- | --- | --- | --- |
| **Overall complications** | 480 (64.5) | 309 (61.8) | 262 (59.5) | 0.2 |
| **Gastrointestinal** |  |  |  |  |
| Anastomotic leaks | 81 (10.9) | 57 (11.4) | 70 (15.9) | **0.026** |
| Grade 1 | 39 (5.2) | 24 (4.8) | 26 (5.9) |  |
| Grade 2 | 25 (3.4) | 15 (3.0) | 24 (5.5) |  |
| Grade 3 | 17 (2.3) | 18 (3.6) | 20 (4.5) |  |
| Conduit necrosis | 9 (1.2) | 12 (2.4) | 17 (3.9) | **0.011** |
| Ileus | 31 (4.2) | 10 (2.0) | 5 (1.1) | **0.004** |
| Small bowel obstruction | 5 (0.7) | 7 (1.4) | 2 (0.5) | 0.2 |
| Feeding J-tube complication | 29 (3.9) | 8 (1.6) | 5 (1.1) | **0.004** |
| Pyloromyotomy/pyloroplasty complication | 4 (0.5) | 3 (0.6) | 1 (0.2) | 0.7 |
| Clostridium difficile Infection | 4 (0.5) | 3 (0.6) | 2 (0.5) | 1.0 |
| Pancreatitis | - | - | - | - |
| GI bleeding requiring intervention or transfusion | 3 (0.4) | 1 (0.2) | 3 (0.7) | 0.5 |
| Liver dysfunction | 3 (0.4) | 1 (0.2) | 3 (0.7) | 0.5 |
| Delayed conduit emptying requiring intervention or delaying discharge or  requiring maintenance of NG drainage >7 days post-op | 23 (3.1) | 27 (5.4) | 23 (5.2) | 0.1 |
| **Pulmonary** |  |  |  |  |
| Overall | 276 (37.1) | 193 (38.6) | 125 (28.4) | **0.002** |
| Pneumonia | 213 (28.6) | 149 (29.8) | 83 (18.9) | **<0.001** |
| Pleural effusion requiring additional drainage procedure | 62 (8.3) | 67 (13.4) | 28 (6.4) | **0.001** |
| Pneumothorax requiring intervention | 10 (1.3) | 10 (2.0) | 13 (3.0) | 0.1 |
| Atelectasis mucous plugging requiring bronchoscopy | 33 (4.4) | 4 (0.8) | 9 (2.0) | **<0.001** |
| Respiratory failure requiring reintubation | 38 (5.1) | 17 (3.4) | 21 (4.8) | 0.3 |
| Acute respiratory distress syndrome | 8 (1.1) | 6 (1.2) | 3 (0.7) | 0.7 |
| Acute aspiration | 8 (1.1) | 3 (0.6) | 5 (1.1) | 0.6 |
| Tracheobronchial injury | 6 (0.8) | 4 (0.8) | 4 (0.9) | 1.0 |
| Chest drain requirement for air leak for >10 days post-operatively | 1 (0.1) | 1 (0.2) | 5 (1.1) | **0.023** |
| **Cardiac** |  |  |  |  |
| Overall | 103 (13.8) | 62 (12.4) | 70 (15.9) | 0.3 |
| Cardiac arrest requiring CPR | 7 (0.9) | 3 (0.6) | 5 (1.1) | 0.7 |
| Myocardial infarction | 6 (0.8) | 2 (0.4) | 1 (0.2) | 0.4 |
| Atrial dysrhythmia requiring intervention | 87 (11.7) | 49 (9.8) | 61 (13.9) | 0.1 |
| Ventricular dysrhythmia requiring intervention | 7 (0.9) | 2 (0.4) | 5 (1.1) | 0.4 |
| Congestive heart failure requiring intervention | 4 (0.5) | 3 (0.6) | 2 (0.5) | 1.0 |
| Pericarditis | - | - | - | - |
| **Thromboembolic** |  |  |  |  |
| Overall | 24 (3.2) | 11 (2.2) | 14 (3.2) | 0.6 |
| Deep vein thrombosis | 8 (1.1) | 6 (1.2) | 4 (0.9) | 0.9 |
| Pulmonary embolism | 19 (2.6) | 3 (0.6) | 9 (2.0) | 0.1 |
| Stroke | 1 (0.1) | 0 (0.0) | 2 (0.5) | 0.2 |
| Peripheral thrombophlebitis | 0 (0.0) | 0 (0.0) | 1 (0.2) | 0.2 |
| **Urologic** |  |  |  |  |
| Overall | 52 (7.0) | 19 (3.8) | 22 (5.0) | 0.1 |
| Acute renal insufficiency (defined as doubling of baseline creatinine) | 21 (2.8) | 8 (1.6) | 5 (1.1) | 0.1 |
| Acute renal failure requiring dialysis | 9 (1.2) | 2 (0.4) | 4 (0.9) | 0.3 |
| Urinary tract infection | 17 (2.3) | 4 (0.8) | 3 (0.7) | **0.03** |
| Urinary retention requiring reinsertion of urinary catheter, delaying  discharge, or discharge with urinary catheter | 8 (1.1) | 4 (0.8) | 13 (3.0) | **0.016** |
| **Infection** |  |  |  |  |
| Overall | 160 (21.5) | 82 (16.4) | 68 (15.5) | **0.016** |
| Wound infection requiring opening wound or antibiotics | 45 (6.0) | 15 (3.0) | 11 (2.5) | **0.004** |
| Central Intravenous line infection requiring removal or antibiotics | 17 (2.3) | 5 (1.0) | 12 (2.7) | 0.1 |
| Intrathoracic/Intra-abdominal abscess | 19 (2.6) | 18 (3.6) | 13 (3.0) | 0.6 |
| Generalized sepsis | 32 (4.3) | 16 (3.2) | 12 (2.7) | 0.3 |
| Other infections requiring antibiotics | 66 (8.9) | 36 (7.2) | 23 (5.2) | 0.1 |
| **Wound / Diaphragm** |  |  |  |  |
| Overall | 14 (1.9) | 10 (2.0) | 6 (1.4) | 0.9 |
| Thoracic wound dehiscence | 10 (1.3) | 3 (0.6) | 1 (0.2) | 0.1 |
| Acute abdominal wall dehiscence/hernia | 2 (0.3) | 0 (0.0) | 2 (0.5) | 0.3 |
| Acute diaphragmatic hernia | 3 (0.4) | 5 (1.0) | 4 (0.9) | 0.4 |
| **Other complications** |  |  |  |  |
| Chyle Leak | 47 (6.3) | 27 (5.4) | 15 (3.4) | 0.1 |
| Type 1 | 19 (2.6) | 9 (1.8) | 3 (0.7) |  |
| Type 2 | 6 (0.8) | 5 (1.0) | 7 (1.6) |  |
| Type 3 | 22 (3.0) | 13 (2.6) | 5 (1.1) |  |
| Return to theatre | 81 (10.9) | 57 (11.4) | 57 (13.0) | 0.5 |
| Length of stay | 12.5 (10.0 - 19.0) | 12.0 (9.0 - 18.0) | 11.0 (8.0 - 15.0) | **<0.001** |

Supplementary Table 9 Hospital-, patient-, and operative-level clinicopathologic characteristics of patients with esophageal cancers receiving open, hybrid and totally minimally invasive esophagectomy in patients with respiratory disease

|  |  | **Open,**  **n=90** | **Hybrid,**  **n=50** | **Totally minimally invasive,**  **n=64** | | **p-value** | |  |
| --- | --- | --- | --- | --- | --- | --- | --- | --- |
| **Hospital-level factors** |  |  |  |  | |  | |  |
| Center volume | <28 | 25 (27.8) | 17 (34.0) | 30 (46.9) | | 0.004 | |  |
|  | 28-50 | 46 (51.1) | 19 (38.0) | 13 (20.3) | |  | |  |
|  | ≥51 | 19 (21.1) | 14 (28.0) | 21 (32.8) | |  | |  |
| Country income | High income country | 86 (95.6) | 49 (98.0) | 59 (92.2) | | 0.349 | |  |
|  | Low-Middle income country | 4 (4.4) | 1 (2.0) | 5 (7.8) | |  | |  |
| **Patient-level factors** |  |  |  |  | |  | |  |
| Age at surgery |  | 65.6 (7.5) | 64.7 (9.5) | 66.0 (9.2) | | 0.706 | |  |
| Sex | Female | 22 (24.4) | 2 (4.0) | 4 (6.2) | | <0.001 | |  |
|  | Male | 68 (75.6) | 48 (96.0) | 60 (93.8) | |  | |  |
| ASA Grade | 1 | 0 (0.0) | 4 (8.0) | 3 (4.7) | | 0.073 | |  |
|  | 2 | 36 (40.0) | 22 (44.0) | 21 (32.8) | |  | |  |
|  | 3-4 | 54 (60.0) | 24 (48.0) | 40 (62.5) | |  | |  |
| Smoking status | Never smoker | 18 (20.0) | 8 (16.0) | 9 (14.1) | | 0.313 | |  |
|  | Ex-smoker | 52 (57.8) | 24 (48.0) | 35 (54.7) | |  | |  |
|  | Current smoker | 17 (18.9) | 17 (34.0) | 20 (31.2) | |  | |  |
|  | Unknown | 3 (3.3) | 1 (2.0) | 0 (0.0) | |  | |  |
| Body Mass Index, kg/m^2^ | ≤18.5 | 3 (3.3) | 2 (4.0) | 0 (0.0) | | 0.580 | |  |
|  | 18.6-24.9 | 31 (34.4) | 22 (44.0) | 22 (34.4) | |  | |  |
|  | 25.0-29.9 | 28 (31.1) | 14 (28.0) | 24 (37.5) | |  | |  |
|  | ≥30.0 | 28 (31.1) | 12 (24.0) | 18 (28.1) | |  | |  |
| Tumor Histology | Adenocarcinoma | 76 (84.4) | 44 (88.0) | 55 (85.9) | | 0.846 | |  |
|  | Squamous Cell Carcinoma | 14 (15.6) | 6 (12.0) | 9 (14.1) | |  | |  |
| Tumor location | Proximal/Middle | 6 (6.7) | 3 (6.0) | 4 (6.2) | | 0.583 | |  |
|  | Distal | 34 (37.8) | 11 (22.0) | 23 (35.9) | |  | |  |
|  | Siewert 1 | 30 (33.3) | 22 (44.0) | 25 (39.1) | |  | |  |
|  | Siewert 2-3 | 20 (22.2) | 14 (28.0) | 12 (18.8) | |  | |  |
| Clinical AJCC T Stage | cT1 | 11 (12.2) | 5 (10.0) | 14 (21.9) | | 0.383 | |  |
|  | cT2 | 22 (24.4) | 12 (24.0) | 12 (18.8) | |  | |  |
|  | cT3/T4a | 57 (63.3) | 33 (66.0) | 38 (59.4) | |  | |  |
| Clinical AJCC N Stage | cN0 | 39 (43.3) | 19 (38.0) | 30 (46.9) | | 0.744 | |  |
|  | cN1 | 38 (42.2) | 20 (40.0) | 23 (35.9) | |  | |  |
|  | cN2/3 | 13 (14.4) | 11 (22.0) | 11 (17.2) | |  | |  |
| Preoperative nutrition | No | 53 (58.9) | 20 (40.0) | 33 (51.6) | | 0.100 | |  |
|  | Yes | 37 (41.1) | 30 (60.0) | 31 (48.4) | |  | |  |
| **Operative-level factors** |  |  |  | |  | |  | |
| Neoadjuvant Therapy | None | 24 (26.7) | 11 (22.0) | | 28 (43.8) | | 0.001 | |
|  | Chemoradiotherapy | 20 (22.2) | 15 (30.0) | | 24 (37.5) | |  | |
|  | Chemotherapy | 46 (51.1) | 24 (48.0) | | 12 (18.8) | |  | |
| Anastomosis technique | Handsewn | 22 (24.4) | 5 (10.0) | | 9 (14.1) | | 0.086 | |
|  | Linear Stapled | 10 (11.1) | 7 (14.0) | | 14 (21.9) | |  | |
|  | Circular stapled | 58 (64.4) | 38 (76.0) | | 41 (64.1) | |  | |
| Gastric tube | Thin (<5cm) | 37 (41.1) | 33 (66.0) | | 50 (78.1) | | <0.001 | |
|  | Wide (>5cm) | 52 (57.8) | 17 (34.0) | | 12 (18.8) | |  | |
|  | Whole Stomach | 1 (1.1) | 0 (0.0) | | 2 (3.1) | |  | |
| Pyloric procedures | Not Performed | 49 (54.4) | 37 (74.0) | | 47 (73.4) | | <0.001 | |
|  | Botox | 0 (0.0) | 0 (0.0) | | 7 (10.9) | |  | |
|  | Dilatation | 2 (2.2) | 9 (18.0) | | 6 (9.4) | |  | |
|  | Pyloromyotomy | 2 (2.2) | 1 (2.0) | | 1 (1.6) | |  | |
|  | Pyloroplasty | 37 (41.1) | 3 (6.0) | | 3 (4.7) | |  | |
| Omentoplasty | No | 67 (74.4) | 27 (54.0) | | 26 (40.6) | | <0.001 | |
|  | Yes | 23 (25.6) | 23 (46.0) | | 38 (59.4) | |  | |

Supplementary Table 10 Univariable and multivariable analysis of postoperative (i.e. pulmonary complications, anastomotic leaks, overall & major complications, and 90-day mortality) outcomes

of patients with esophageal cancers receiving open, hybrid and totally minimally invasive esophagectomy with respiratory disease

|  | **Rates, n (%)** | **Univariable, OR (95% CI)** | **Multivariable, OR (95% CI)** |
| --- | --- | --- | --- |
| **Pulmonary complications*** |  |  |  |
| Open | 44 (48.9) | REF | REF |
| Hybrid | 22 (44.0) | 1.13 (0.89-1.45, p=0.318) | 1.10 (0.85-1.42, p=0.486) |
| Totally minimally invasive | 30 (46.9) | 0.62 (0.47-0.82, p=0.001) | 0.53 (0.39-0.72, p<0.001) |
|  |  |  |  |
| **Overall complications*** |  |  |  |
| Open | 72 (80.0) | REF | REF |
| Hybrid | 34 (68.0) | 0.53 (0.24-1.17, p=0.115) | 0.46 (0.16-1.26, p=0.132) |
| Totally minimally invasive | 46 (71.9) | 0.64 (0.30-1.36, p=0.242) | 0.85 (0.33-2.20, p=0.735) |
|  |  |  |  |
| **Major complications*** |  |  |  |
| Open | 26 (28.9) | REF | REF |
| Hybrid | 12 (24.0) | 0.78 (0.34-1.69, p=0.534) | 0.63 (0.25-1.52, p=0.309) |
| Totally minimally invasive | 25 (39.1) | 1.58 (0.80-3.12, p=0.187) | 1.44 (0.62-3.37, p=0.395) |
|  |  |  |  |
| **Anastomotic leaks*** |  |  |  |
| Open | 21 (23.3) | REF | REF |
| Hybrid | 4 (8.0) | 0.29 (0.08-0.81, p=0.030) | 0.21 (0.05-0.68, p=0.015) |
| Totally minimally invasive | 18 (28.1) | 1.29 (0.61-2.68, p=0.501) | 1.05 (0.41-2.69, p=0.919) |
|  |  |  |  |
| **90-day mortality*** |  |  |  |
| Open | 2 (2.2) | REF | REF |
| Hybrid | 1 (2.0) | 0.90 (0.04-9.60, p=0.931) | - |
| Totally minimally invasive | 4 (6.2) | 2.93 (0.55-21.64, p=0.222) | - |

**Adjusted for center volume, country income, age at surgery, sex, ASA grade, smoking status, body mass index, tumor histology & location, clinical AJCC T stage, clinical AJCC N stage, preoperative nutrition, neoadjuvant therapy, anastomotic technique, and surgical approach*

Supplementary Table 11 Hospital-, patient-, and operative-level clinicopathologic characteristics of patients with esophageal cancers receiving open, hybrid and totally minimally invasive esophagectomy in patients with neoadjuvant chemoradiotherapy

|  |  | | **Open,**  **n=201** | | **Hybrid,**  **n=114** | **Totally minimally invasive,**  **n=184** | **p-value** |
| --- | --- | --- | --- | --- | --- | --- | --- |
| **Hospital-level factors** |  | |  | |  |  |  |
| Center volume | <28 | | 104 (51.7) | | 32 (28.1) | 62 (33.7) | <0.001 |
|  | 28-50 | | 66 (32.8) | | 47 (41.2) | 61 (33.2) |  |
|  | ≥51 | | 31 (15.4) | | 35 (30.7) | 61 (33.2) |  |
| Country income | High income country | | 194 (96.5) | | 112 (98.2) | 183 (99.5) | 0.118 |
|  | Low-Middle income country | | 7 (3.5) | | 2 (1.8) | 1 (0.5) |  |
| **Patient-level factors** |  | |  | |  |  |  |
| Age at surgery |  | | 64.2 (9.9) | | 65.1 (9.1) | 63.8 (9.1) | 0.532 |
| Sex | Female | | 53 (26.4) | | 22 (19.3) | 39 (21.2) | 0.285 |
|  | Male | | 148 (73.6) | | 92 (80.7) | 145 (78.8) |  |
| ASA Grade | 1 | | 15 (7.5) | | 15 (13.2) | 9 (4.9) | 0.018 |
|  | 2 | | 116 (57.7) | | 64 (56.1) | 92 (50.0) |  |
|  | 3-4 | | 70 (34.8) | | 35 (30.7) | 83 (45.1) |  |
| Smoking status | Never smoker | | 73 (36.3) | | 31 (27.2) | 56 (30.4) | 0.075 |
|  | Ex-smoker | | 94 (46.8) | | 62 (54.4) | 101 (54.9) |  |
|  | Current smoker | | 26 (12.9) | | 16 (14.0) | 27 (14.7) |  |
|  | Unknown | | 8 (4.0) | | 5 (4.4) | 0 (0.0) |  |
| Respiratory disease | No | | 181 (90.0) | | 99 (86.8) | 160 (87.0) | 0.567 |
|  | Yes | | 20 (10.0) | | 15 (13.2) | 24 (13.0) |  |
| Body mass index | ≤18.5 | | 13 (6.5) | | 5 (4.4) | 2 (1.1) | 0.010 |
|  | 18.6-24.9 | | 98 (48.8) | | 46 (40.4) | 68 (37.0) |  |
|  | 25.0-29.9 | | 55 (27.4) | | 44 (38.6) | 75 (40.8) |  |
|  | ≥30.0 | | 35 (17.4) | | 19 (16.7) | 39 (21.2) |  |
| Tumor Histology | Adenocarcinoma | | 124 (61.7) | | 68 (59.6) | 155 (84.2) | <0.001 |
|  | Squamous Cell Carcinoma | | 77 (38.3) | | 46 (40.4) | 29 (15.8) |  |
| Tumor location | Proximal/Middle | | 38 (18.9) | | 20 (17.5) | 9 (4.9) | <0.001 |
|  | Distal | | 84 (41.8) | | 36 (31.6) | 101 (54.9) |  |
|  | Siewert 1 | | 52 (25.9) | | 40 (35.1) | 47 (25.5) |  |
|  | Siewert 2-3 | | 27 (13.4) | | 18 (15.8) | 27 (14.7) |  |
| Clinical AJCC T Stage | cT1 | | 16 (8.0) | | 4 (3.5) | 10 (5.4) | 0.522 |
|  | cT2 | | 35 (17.4) | | 19 (16.7) | 28 (15.2) |  |
|  | cT3/T4a | | 150 (74.6) | | 91 (79.8) | 146 (79.3) |  |
| Clinical AJCC N Stage | cN0 | | 68 (33.8) | | 39 (34.2) | 66 (35.9) | 0.410 |
|  | cN1 | | 91 (45.3) | | 46 (40.4) | 88 (47.8) |  |
|  | cN2/3 | | 42 (20.9) | | 29 (25.4) | 30 (16.3) |  |
| Preoperative nutrition | No | | 85 (42.3) | | 46 (40.4) | 84 (45.7) | 0.640 |
|  | Yes | | 116 (57.7) | | 68 (59.6) | 100 (54.3) |  |
| **Operative-level factors** |  |  | |  | |  |  |
| Anastomosis technique | Handsewn | 25 (12.4) | | 9 (7.9) | | 36 (19.6) | <0.001 |
|  | Linear Stapled | 25 (12.4) | | 21 (18.4) | | 48 (26.1) |  |
|  | Circular stapled | 151 (75.1) | | 84 (73.7) | | 100 (54.3) |  |
| Gastric tube | Thin (<5cm) | 84 (41.8) | | 71 (62.3) | | 136 (73.9) | <0.001 |
|  | Wide (>5cm) | 116 (57.7) | | 41 (36.0) | | 48 (26.1) |  |
|  | Whole Stomach | 1 (0.5) | | 2 (1.8) | | 0 (0.0) |  |
| Pyloric procedures | Not Performed | 105 (52.2) | | 79 (69.3) | | 147 (79.9) | <0.001 |
|  | Botox | 4 (2.0) | | 0 (0.0) | | 17 (9.2) |  |
|  | Dilatation | 8 (4.0) | | 23 (20.2) | | 9 (4.9) |  |
|  | Pyloromyotomy | 7 (3.5) | | 1 (0.9) | | 1 (0.5) |  |
|  | Pyloroplasty | 77 (38.3) | | 11 (9.6) | | 10 (5.4) |  |
| Omentoplasty | No | 129 (64.2) | | 54 (47.4) | | 62 (33.7) | <0.001 |
|  | Yes | 72 (35.8) | | 60 (52.6) | | 122 (66.3) |  |

Supplementary Table 12 Univariable and multivariable analysis of postoperative (i.e. pulmonary complications, anastomotic leaks, overall & major complications, and 90-day mortality) outcomes

of patients with esophageal cancers receiving open, hybrid and totally minimally invasive esophagectomy with neoadjuvant chemoradiotherapy

|  | **Rates, n (%)** | **Univariable, OR (95% CI)** | **Multivariable, OR (95% CI)** |
| --- | --- | --- | --- |
| **Pulmonary complications*** |  |  |  |
| Open | 75 (37.3) | REF | REF |
| Hybrid | 43 (37.7) | 1.02 (0.63-1.63, p=0.943) | 0.90 (0.54-1.51, p=0.695) |
| Totally minimally invasive | 54 (29.3) | 0.70 (0.45-1.07, p=0.099) | 0.59 (0.36-0.96, p=0.035) |
|  |  |  |  |
| **Overall complications*** |  |  |  |
| Open | 136 (67.7) | REF | REF |
| Hybrid | 69 (60.5) | 0.73 (0.45-1.18, p=0.202) | 0.61 (0.36-1.05, p=0.074) |
| Totally minimally invasive | 120 (65.2) | 0.90 (0.59-1.37, p=0.612) | 0.79 (0.48-1.30, p=0.353) |
|  |  |  |  |
| **Major complications*** |  |  |  |
| Open | 57 (28.4) | REF | REF |
| Hybrid | 32 (28.1) | 0.99 (0.59-1.64, p=0.956) | 0.88 (0.51-1.53, p=0.665) |
| Totally minimally invasive | 46 (25.0) | 0.84 (0.53-1.32, p=0.457) | 0.72 (0.43-1.20, p=0.214) |
|  |  |  |  |
| **Anastomotic leaks*** |  |  |  |
| Open | 21 (10.4) | REF | REF |
| Hybrid | 14 (12.3) | 1.20 (0.57-2.44, p=0.619) | 1.02 (0.46-2.21, p=0.966) |
| Totally minimally invasive | 31 (16.8) | 1.74 (0.96-3.18, p=0.069) | 1.75 (0.88-3.55, p=0.113) |
|  |  |  |  |
| **90-day mortality*** |  |  |  |
| Open | 10 (5.0) | REF | REF |
| Hybrid | 4 (3.5) | 0.69 (0.19-2.13, p=0.546) | 0.38 (0.08-1.47, p=0.185) |
| Totally minimally invasive | 5 (2.7) | 0.53 (0.16-1.53, p=0.260) | 0.34 (0.08-1.30, p=0.127) |

**Adjusted for center volume, country income, age at surgery, sex, ASA grade, smoking status, respiratory disease, body mass index, tumor histology & location, clinical AJCC T stage, clinical AJCC N stage, preoperative nutrition, anastomotic technique, and surgical approach*

Supplementary Table 13 Patient-, and operative-level clinicopathologic characteristics of patients with esophageal cancers receiving open, hybrid and totally minimally invasive esophagectomy in high volume centers

|  |  | | **Open,**  **n=205** | | **Hybrid,**  **n=166** | **Totally minimally invasive,**  **n=156** | **p-value** |
| --- | --- | --- | --- | --- | --- | --- | --- |
| **Patient-level factors** |  | |  | |  |  |  |
| Age at surgery |  | | 64.7 (9.4) | | 66.0 (9.5) | 64.4 (9.8) | 0.242 |
| Sex | Female | | 40 (19.5) | | 24 (14.5) | 32 (20.5) | 0.308 |
|  | Male | | 165 (80.5) | | 142 (85.5) | 124 (79.5) |  |
| ASA Grade | 1 | | 23 (11.2) | | 25 (15.1) | 12 (7.7) | 0.251 |
|  | 2 | | 126 (61.5) | | 93 (56.0) | 103 (66.0) |  |
|  | 3-4 | | 56 (27.3) | | 48 (28.9) | 41 (26.3) |  |
| Smoking status | Never smoker | | 84 (41.0) | | 52 (31.3) | 45 (28.8) | 0.023 |
|  | Ex-smoker | | 88 (42.9) | | 73 (44.0) | 81 (51.9) |  |
|  | Current smoker | | 31 (15.1) | | 34 (20.5) | 29 (18.6) |  |
|  | Unknown | | 2 (1.0) | | 7 (4.2) | 1 (0.6) |  |
| Respiratory disease | No | | 186 (90.7) | | 152 (91.6) | 135 (86.5) | 0.278 |
|  | Yes | | 19 (9.3) | | 14 (8.4) | 21 (13.5) |  |
| Body mass index | ≤18.5 | | 3 (1.5) | | 4 (2.4) | 3 (1.9) | 0.295 |
|  | 18.6-24.9 | | 72 (35.1) | | 59 (35.5) | 58 (37.2) |  |
|  | 25.0-29.9 | | 72 (35.1) | | 68 (41.0) | 68 (43.6) |  |
|  | ≥30.0 | | 58 (28.3) | | 35 (21.1) | 27 (17.3) |  |
| Tumor Histology | Adenocarcinoma | | 165 (80.5) | | 139 (83.7) | 140 (89.7) | 0.056 |
|  | Squamous Cell Carcinoma | | 40 (19.5) | | 27 (16.3) | 16 (10.3) |  |
| Tumor location | Proximal/Middle | | 13 (6.3) | | 10 (6.0) | 7 (4.5) | 0.046 |
|  | Distal | | 50 (24.4) | | 45 (27.1) | 59 (37.8) |  |
|  | Siewert 1 | | 83 (40.5) | | 68 (41.0) | 42 (26.9) |  |
|  | Siewert 2-3 | | 59 (28.8) | | 43 (25.9) | 48 (30.8) |  |
| Clinical AJCC T Stage | cT1 | | 17 (8.3) | | 14 (8.4) | 8 (5.1) | 0.175 |
|  | cT2 | | 59 (28.8) | | 40 (24.1) | 31 (19.9) |  |
|  | cT3/T4a | | 129 (62.9) | | 112 (67.5) | 117 (75.0) |  |
| Clinical AJCC N Stage | cN0 | | 92 (44.9) | | 78 (47.0) | 73 (46.8) | 0.881 |
|  | cN1 | | 79 (38.5) | | 57 (34.3) | 53 (34.0) |  |
|  | cN2/3 | | 34 (16.6) | | 31 (18.7) | 30 (19.2) |  |
| Preoperative nutrition | No | | 99 (48.3) | | 72 (43.4) | 56 (35.9) | 0.062 |
|  | Yes | | 106 (51.7) | | 94 (56.6) | 100 (64.1) |  |
| **Operative-level factors** |  |  | |  | |  |  |
| Neoadjuvant Therapy | None | 47 (22.9) | | 37 (22.3) | | 36 (23.1) | <0.001 |
|  | Chemoradiotherapy | 31 (15.1) | | 35 (21.1) | | 61 (39.1) |  |
|  | Chemotherapy | 127 (62.0) | | 94 (56.6) | | 59 (37.8) |  |
| Anastomotic Technique | Handsewn | 10 (4.9) | | 4 (2.4) | | 55 (35.3) | <0.001 |
|  | Linear Stapled | 68 (33.2) | | 36 (21.7) | | 0 (0.0) |  |
|  | Circular stapled | 127 (62.0) | | 126 (75.9) | | 101 (64.7) |  |
| Gastric tube | Thin (<5cm) | 112 (54.6) | | 121 (72.9) | | 124 (79.5) | <0.001 |
|  | Wide (>5cm) | 91 (44.4) | | 45 (27.1) | | 32 (20.5) |  |
|  | Whole Stomach | 2 (1.0) | | 0 (0.0) | | 0 (0.0) |  |
| Pyloric procedures | Not Performed | 75 (36.6) | | 109 (65.7) | | 124 (79.5) | <0.001 |
|  | Botox | 0 (0.0) | | 0 (0.0) | | 0 (0.0) |  |
|  | Dilatation | 17 (8.3) | | 31 (18.7) | | 16 (10.3) |  |
|  | Pyloromyotomy | 0 (0.0) | | 1 (0.6) | | 3 (1.9) |  |
|  | Pyloroplasty | 113 (55.1) | | 25 (15.1) | | 13 (8.3) |  |
| Omentoplasty | No | 118 (57.6) | | 69 (41.6) | | 52 (33.3) | <0.001 |
|  | Yes | 87 (42.4) | | 97 (58.4) | | 104 (66.7) |  |

Supplementary Table 14 Univariable and multivariable analysis of postoperative (i.e. pulmonary complications, anastomotic leaks, overall & major complications, and 90-day mortality) outcomes

of patients with esophageal cancers receiving open, hybrid and totally minimally invasive esophagectomy in high volume centers

|  | **Rates, n (%)** | **Univariable, OR (95% CI)** | **Multivariable, OR (95% CI)** |
| --- | --- | --- | --- |
| **Pulmonary complications*** |  |  |  |
| Open | 70 (34.1) | REF | REF |
| Hybrid | 65 (39.2) | 1.24 (0.81-1.90, p=0.319) | 1.40 (0.87-2.24, p=0.165) |
| Totally minimally invasive | 36 (23.1) | 0.58 (0.36-0.92, p=0.023) | 0.44 (0.23-0.82, p=0.011) |
|  |  |  |  |
| **Overall complications*** |  |  |  |
| Open | 131 (63.9) | REF | REF |
| Hybrid | 117 (70.5) | 1.35 (0.87-2.10, p=0.181) | 1.67 (1.03-2.73, p=0.041) |
| Totally minimally invasive | 83 (53.2) | 0.64 (0.42-0.98, p=0.041) | 0.80 (0.45-1.41, p=0.437) |
|  |  |  |  |
| **Major complications*** |  |  |  |
| Open | 54 (26.3) | REF | REF |
| Hybrid | 49 (29.5) | 1.17 (0.74-1.85, p=0.497) | 1.15 (0.70-1.90, p=0.582) |
| Totally minimally invasive | 27 (17.3) | 0.59 (0.34-0.98, p=0.043) | 0.48 (0.24-0.94, p=0.037) |
|  |  |  |  |
| **Anastomotic leaks*** |  |  |  |
| Open | 18 (8.8) | REF | REF |
| Hybrid | 19 (11.4) | 1.34 (0.68-2.67, p=0.396) | 1.28 (0.60-2.71, p=0.517) |
| Totally minimally invasive | 18 (11.5) | 1.36 (0.68-2.71, p=0.388) | 0.91 (0.34-2.40, p=0.856) |
|  |  |  |  |
| **90-day mortality*** |  |  |  |
| Open | 6 (2.9) | REF | REF |
| Hybrid | 7 (4.2) | 1.46 (0.48-4.62, p=0.504) | 0.95 (0.23-3.91, p=0.938) |
| Totally minimally invasive | 1 (0.6) | 0.21 (0.01-1.27, p=0.155) | 0.28 (0.01-2.81, p=0.334) |

**Adjusted for country income, age at surgery, sex, ASA grade, smoking status, respiratory disease, body mass index, tumor histology & location, clinical AJCC T stage, clinical AJCC N stage, preoperative nutrition, neoadjuvant therapy, anastomotic technique, and surgical approach.*

**OGAA Author List (All to be PubMed citable)**

**Steering Committee**: Alderson D, Bundred J, Evans RPT, Gossage J, Griffiths EA, Jefferies B, Kamarajah SK, McKay S, Mohamed I, Nepogodiev D, Siaw- Acheampong K, Singh P, van Hillegersberg R, Vohra R, Wanigasooriya K, Whitehouse T.

**National Leads:** Gjata A (Albania), Moreno JI (Argentina), Takeda FR (Brazil), Kidane B (Canada), Guevara Castro R (Colombia), Harustiak T (Czech Republic), Bekele A (Ethiopia), Kechagias A (Finland), Gockel I (Germany), Kennedy A (Ireland), Da Roit A (Italy), Bagajevas A (Lithuania), Azagra JS (Luxembourg), Mahendran HA (Malaysia), Mejía-Fernández L (Mexico), Wijnhoven BPL (Netherlands), El Kafsi J (New Zealand), Sayyed RH (Pakistan), Sousa M (Portugal), Sampaio AS (Portugal), Negoi I (Romania), Blanco R (Spain), Wallner B (Sweden), Schneider PM (Switzerland), Hsu PK (Taiwan), Isik A (Turkey)

**Site Leads:**

Gananadha S (The Canberra Hospital, Australia); Wills V (John Hunter Hospital, Australia); Devadas M (Nepean Hospital, Australia); Duong C (Peter MacCallum Cancer Centre, Australia); Talbot M (St George Public and Private Hospitals, Australia); Hii MW (St Vincent's Hospital Melbourne, Australia); Jacobs R (Western Hospital, Victoria, Australia); Andreollo NA (Unicamp University Hospital, Brazil); Johnston B (Saint John Regional Hospital, Canada); Darling G (Toronto General Hospital, University Health Network, Canada); Isaza-Restrepo A (Hospital Universitario Mayor Mederi-Universidad del Rosario, Colombia); Rosero G (Hospital San Ignacio-Universidad Javeriana, Colombia); Arias- Amézquita F (University Hospital Fundacion Santafe de Bogota, Colombia); Raptis D (University Clinic of Erlangen, Germany); Gaedcke J (Medical Unversity Goettingen, Germany); Reim D (Klinikum Rechts der Isar der TU München, Germany); Izbicki J (University Hospital Hamburg Eppendorf, Germany); Egberts JH(University Hospital Kiel, Germany); Dikinis S (Aalborg University Hospital, Denmark); Kjaer DW (Aarhus University Hospital, Denmark); Larsen MH (Odense University Hospital, Denmark); Achiam MP (Copenhagen University hospital Rigshospitalet, Denmark); Saarnio J (Oulu University Hospital, Finland); Theodorou D (Hippokration General Hospital University of Athens, Greece); Liakakos T (Laikon General Hospital, Greece); Korkolis DP (St. Savvas Cancer Hospital, Greece); Robb WB (Beaumont Hospital, Ireland); Collins C (University Hospital Galway, Ireland); Murphy T (Mercy University Hospital, Ireland); Reynolds J (St James's Hospital, Dublin, Ireland); Tonini V (St. Orsola Hospital- University of Bologna, Italy); Migliore M (Polyclinic Hospital University of Catania, Italy); Bonavina L (University of Milano, IRCCS Policlinico San Donato, Department of General and Foregut Surgery, Italy); Valmasoni M (Padova University Hospital - Clinica Chirurgica 3, Italy); Bardini R (Padova University Hospital- General Surgery Department, Italy); Weindelmayer J (Verona Borgo Trento Hospital, Italy); Terashima M (Shizioka Cancer Centre, Japan); White RE (Tenwek Hospital, Kenya); Alghunaim E (Chest Diseases Hospital, Kuwait); Elhadi M (Tripoli, Libya); Leon-Takahashi AM (National Cancer Institute, Mexico); Medina-Franco H (National Institute of Medical Science and Nutrition Salvador Zubirán, Mexico); Lau PC (University Malaya Medical Centre, Malaysia); Okonta KE (Carez Hospital & University of Port-Harcourt Teaching Hospital, Nigeria); Heisterkamp J (Elisabeth-TweeSteden Ziekenhuis Hospital, Netherlands); Rosman C (Radboudumc, Netherlands); van Hillegersberg R (UMC Utrecht, Netherlands); Beban G (Auckland City Hospital, New Zealand); Babor R (Middlemore Hospital, New Zealand); Gordon A (Palmerston North Hospital, New Zealand); Rossaak JI (Tauranga Hospital, Bay of Plenty District Health Board, New Zealand); Pal KMI (Aga Khan University Hospital, Pakistan); Qureshi AU (Services Institute of Medical Sciences, Lahore, Pakistan); Naqi SA (Mayo Hospital, Lahore, Pakistan); Syed AA (Shaukat Khanum Memorial Cancer Hospital & Research Centre Lahore, Pakistan); Barbosa J (Centro Hospitalar São João, Portugal); Vicente CS (Centro Hospitalar Lisboa Central, Portugal); Leite J (Coimbra University Hospital, Portugal); Freire J (Hospital Santa Maria, Portugal); Casaca R (Instituto Português de Oncologia de Lisboa, Portugal); Costa RCT (Instituto Português de Oncologia do Porto, Portugal); Scurtu RR (University Emergency Cluj County Hospital, Romania); Mogoanta SS (Emergency County Hospital of Craiova, Romania); Bolca C (Marius Nasta' National Institute of Pneumology, Romania); Constantinoiu S (St. Mary Clinical Hospital, Romania); Sekhniaidze D (Tyumen Regional Hospital, Russia); Bjelović M (Department for Minimally Invasive Upper Digestive Surgery, University Hospital for Digestive Surgery, Clinical Center of Serbia, Belgrade, Serbia); So JBY (National University Hospital, Singapore); Gačevski G (University Hospital Maribor, Slovenia); Loureiro C (University Hospital of Basurto (Bilbao), Spain); Pera M (Hospital Universitario del Mar, Spain); Bianchi A (Palma de Mallorca, Spain); Moreno Gijón M (Hospital Universitario Central de Asturias, Spain); Martín Fernández J (Hospital General Universitario De Ciudad Real, Spain); Trugeda Carrera MS (Hospital Universitario Marqués de Valdecilla, Spain); Vallve-Bernal M (Hospital Universitario Nuestra Señora de Candelaria, Spain); Cítores Pascual MA (Hospital Universitario Río Hortega de Valladolid, Spain); Elmahi S (Shaab Teaching Hospital, Sudan), Halldestam I (University Hospital Linköping, Sweden); Hedberg J (Uppsala University Hospital, Sweden); Mönig S (Geneva University Hospital, Switzerland); Gutknecht S (Triemli Hospital Zurich, Switzerland); Tez M (Ankara Numune Hospital, Turkey); Guner A (Karadeniz Technical University, Turkey); Tirnaksiz MB (Hacettepe University Hospital, Turkey); Colak E (University of Health Sciences, Samsun Training and Research Hospital, Turkey); Sevinç B (Usak University Training and Research Hospital, Turkey); Hindmarsh A (Addenbrooke's Hospital, Cambridge, United Kingdom (UK)); Khan I (Aintree University Hospital, Liverpool, UK); Khoo D (Barking Havering and Redbridge NHS Trust, UK); Byrom R (Royal Bournemouth Hospital, UK); Gokhale J (Bradford Royal Infirmary, UK); Wilkerson P (University Hospitals Bristol NHS Foundation Trust, UK); Jain P (Castle Hill Hospital, UK); Chan D (University Hospital of Coventry, UK); Robertson K (University Hospital Crosshouse, UK); Iftikhar S (Royal Derby Hospital, UK); Skipworth R (Edinburgh Royal Infirmary, UK); Forshaw M (Glasgow Royal Infirmary, UK); Higgs S (Gloucester Royal Hospital, UK); Gossage J (Guy's and St Thomas's Hospitals, UK); Nijjar R (Heartlands Hospital, UK); Viswanath YKS (James Cook University Hospital, UK); Turner P (Lancashire Teaching Hospitals NHS Foundation Trust, UK); Dexter S (Leeds Teaching Hospitals NHS Trust, UK); Boddy A (University Hospitals of Leicester NHS Trust, UK); Allum WH (Royal Marsden Hospital, UK); Oglesby S (Ninewells Hospital, UK); Cheong E (Norfolk and Norwich University Hospital, UK); Beardsmore D (University Hospital of North Midlands, UK); Vohra R (Nottingham University Hospital, UK); Maynard N (Oxford University Hospitals, UK); Berrisford R (Plymouth Hospitals NHS Trust, UK); Mercer S (Queen Alexandra Hospital, Portsmouth, UK); Puig S (Queen Elizabeth Hospital Birmingham, UK); Melhado R (Salford Royal Foundation Trust, UK); Kelty C (Sheffield Teaching Hospitals NHS Foundation Trust, UK); Underwood T (University Hospital Southampton NHS Foundation Trust, UK); Dawas K (University College Hospital, UK); Lewis W (University Hospital of Wales, UK); Al-Bahrani A (Watford General Hospital); Bryce G (University Hospital Wishaw, UK); Thomas M (Mayo Clinic in Florida, United States of America (USA)); Arndt AT (Rush University Medical Center, USA); Palazzo F (Thomas Jefferson University, USA); Meguid RA (University of Colorado Hospital, USA)

**Collaborators:**

Fergusson J, Beenen E, Mosse C, Salim J (The Canberra Hospital, Australia); Cheah S, Wright T, Cerdeira MP, McQuillan P (John Hunter Hospital, Australia); Richardson M, Liem H ( Nepean Hospital, Australia); Spillane J, Yacob M, Albadawi F, Thorpe T, Dingle A, Cabalag C (Peter MacCallum Cancer Centre, Australia); Loi K, Fisher OM (St George Public and Private Hospitals, Australia); Ward S, Read M, Johnson M (St Vincent's Hospital Melbourne, Australia); Bassari R, Bui H (Western Hospital, Victoria); Cecconello I, Sallum RAA, da Rocha JRM (Hospital das Clinicas, University of Sao Paulo School of Medicine, Brazil); Lopes LR, Tercioti V Jr, Coelho JDS, Ferrer JAP (Unicamp University Hospital, Brazil); Buduhan G, Tan L, Srinathan S (Health Sciences Centre (Winnipeg)); Shea P (Saint John Regional Hospital, Canada); Yeung J, Allison F, Carroll P (Toronto General Hospital, University Health Network, Canada); Vargas-Barato F, Gonzalez F, Ortega J, Nino-Torres L, Beltrán-García TC (Hospital Universitario Mayor Mederi-Universidad del Rosario, Colombia); Castilla L, Pineda M (Hospital San Ignacio-Universidad Javeriana, Colombia); Bastidas A, Gómez-Mayorga J, Cortés N, Cetares C, Caceres S, Duarte S (University Hospital Fundacion Santafe de Bogota, Colombia); Pazdro A, Snajdauf M, Faltova H, Sevcikova M (Motol University Hospital, Prague, Czech Republic); Mortensen PB (Aalborg University Hospital, Denmark); Katballe N, Ingemann T, Morten B, Kruhlikava I (Aarhus University Hospital, Denmark); Ainswort AP, Stilling NM, Eckardt J (Odense University Hospital, Denmark); Holm J, Thorsteinsson M, Siemsen M, Brandt B (Copenhagen University hospital Rigshospitalet, Denmark); Nega B, Teferra E, Tizazu A (Tikur Anbessa Specialized Hospital, Ethiopa); Kauppila JH, Koivukangas V, Meriläinen S (Oulu University Hospital, Finland); Gruetzmann R, Krautz C, Weber G, Golcher H (University Clinic of Erlangen, Germany); Emons G, Azizian A, Ebeling M (Medical University Goettingen, Germany); Niebisch S, Kreuser N, Albanese G, Hesse J (Universitätklinium Leipzig, Germany); Volovnik L, Boecher U (Klinikum Rechts der Isar der TU München, Germany); Reeh M (University Hospital Hamburg Eppendorf, Germany); Triantafyllou S (Hippokration General Hospital University of Athens, Greece); Schizas D, Michalinos A, Balli E, Mpoura M, Charalabopoulos A (Laikon General Hospital, Greece); Manatakis DK, Balalis D (St. Savvas Cancer Hospital, Greece); Bolger J, Baban C, Mastrosimone A (Beaumont Hospital, Ireland); McAnena O, Quinn A (University Hospital Galway, Ireland); Ó Súilleabháin CB, Hennessy MM, Ivanovski I, Khizer H (Mercy University Hospital, Ireland); Ravi N, Donlon N (St James's Hospital, Dublin, Ireland); Cervellera M, Vaccari S, Bianchini S, Sartarelli l (St. Orsola Hospital- University of Bologna, Italy); Asti E, Bernardi D (University of Milano, IRCCS Policlinico San Donato, Department of General and Foregut Surgery, Italy); Merigliano S, Provenzano L (Padova University Hospital - Clinica Chirurgica, Italy); Scarpa M, Saadeh L, Salmaso B (Padova University Hospital- General Surgery Department, Italy); De Manzoni G, Giacopuzzi S, La Mendola R, De Pasqual CA (Verona Borgo Trento Hospital, Italy); Tsubosa Y, Niihara M, Irino T, Makuuchi R, Ishii K (Shizioka Cancer Centre, Japan); Mwachiro M, Fekadu A, Odera A, Mwachiro E (Tenwek Hospital, Kenya); AlShehab D (Chest diseases hospital, Kuwait); Ahmed HA, Shebani AO, Elhadi A, Elnagar FA, Elnagar HF (Tripoli, Libya); Makkai-Popa ST (Centre Hospitalier de Luxembourg, Luxembourg); Wong LF (University Malaya Medical Centre, Malaysia); Tan YR, Thannimalai S, Ho CA, Pang WS, Tan JH (Hospital Sultanah Aminah, Malaysia); Basave HNL (National Cancer Institute, Mexico); Cortés-González R (Instituto Nacional de Ciencias Médicas y Nutrición 'Salvador Zubirán', Mexico); Lagarde SM, van Lanschot JJB, Cords C (Erasmus University Medical Center, Rotterdam, Netherlands); Jansen WA, Martijnse I, Matthijsen R (Elisabeth-TweeSteden Ziekenhuis Hospital, Netherlands); Bouwense S, Klarenbeek B, Verstegen M, van Workum F (Radboudumc, Netherlands); Ruurda JP, van der Sluis PC, de Maat M (UMC Utrecht, Netherlands); Evenett N, Johnston P, Patel R (Auckland City Hospital, New Zealand); MacCormick A (Middlemore Hospital, New Zealand); Young M (Palmerston North Hospital); Smith B (Tauranga Hospital, Bay of Plenty District Health Board, New Zealand); Ekwunife C (Carez Hospital & University of Port-Harcourt Teaching Hospital, Nigeria); Memon AH, Shaikh K, Wajid A (Aga Khan University Hospital, Pakistan); Khalil N, Haris M, Mirza ZU, Qudus SBA (Services Institute of Medical Sciences, Lahore, Pakistan); Sarwar MZ, Shehzadi A, Raza A, Jhanzaib MH (Mayo Hospital, Lahore, Pakistan); Farmanali J, Zakir Z (Patel Hospital, Pakistan); Shakeel O, Nasir I, Khattak S, Baig M, Noor MA, Ahmed HH, Naeem A (Shaukat Khanum Memorial Cancer Hospital & Research Centre Lahore, Pakistan); Pinho AC, da Silva R (Centro Hospitalar Lisboa Central, Portugal), Bernardes A, Campos JC (Coimbra University Hospital, Portugal); Matos H, Braga T (Hospital Santa Maria, Portugal); Monteiro C, Ramos P, Cabral F (Instituto Português de Oncologia de Lisboa, Portugal); Gomes MP, Martins PC, Correia AM, Videira JF (Instituto Português de Oncologia do Porto, Portugal); Ciuce C, Drasovean R, Apostu R, Ciuce C (University Emergency Cluj County Hospital, Romania); Paitici S, Racu AE, Obleaga CV (Emergency County Hospital of Craiova, Romania); Beuran M, Stoica B, Ciubotaru C, Negoita V (Emergency Hospital of Bucharest, Romania); Cordos I (Marius Nasta' National Institute of Pneumology, Romania); Birla RD, Predescu D, Hoara PA, Tomsa R (St. Mary Clinical Hospital, Romania); Shneider V, Agasiev M, Ganjara I (Tyumen Regional Hospital, Russia); Gunjić D, Veselinović M, Babič T (Department for Minimally Invasive Upper Digestive Surgery, University Hospital for Digestive Surgery, Clinical Center of Serbia, Belgrade, Serbia); Chin TS, Shabbir A, Kim G (National University Hospital, Singapore); Crnjac A, Samo H (University Hospital Maribor, Slovenia); Díez del Val I, Leturio S (University Hospital of Basurto (Bilbao), Spain); Ramón JM, Dal Cero M, Rifá S, Rico M (Hospital Universitario del Mar, Spain); Pagan Pomar A, Martinez Corcoles JA (Palma de Mallorca, Spain); Rodicio Miravalles JL, Pais SA, Turienzo SA, Alvarez LS (Hospital Universitario Central de Asturias, Spain); Campos PV, Rendo AG, García SS, Santos EPG (Hospital General Universitario De Ciudad Real, Spain); Martínez ET, Fernández Díaz MJ, Magadán Álvarez C (Hospital Universitario Marqués de Valdecilla, Spain); Concepción Martín V, Díaz López C, Rosat Rodrigo A, Pérez Sánchez LE (Hospital Universitario Nuestra Señora de Candelaria, Spain); Bailón Cuadrado M, Tinoco Carrasco C, Choolani Bhojwani E, Sánchez DP (Hospital Universitario Río Hortega de Valladolid, Spain); Ahmed ME (Shaab Teaching Hospital, Sudan); Dzhendov T (University Hospital Linköping, Sweden); Lindberg F, Rutegård M (Umeå University Hospital, Sweden); Sundbom M (Uppsala University Hospital, Sweden); Mickael C, Colucci N (Geneva University Hospital, Switzerland); Schnider A (Triemli Hospital Zurich, Switzerland); Er S (Ankara Numune Hospital, Turkey); Kurnaz E (Erzincan University Hospital, Turkey); Turkyilmaz S, Turkyilmaz A, Yildirim R, Baki BE (Karadeniz Technical University, Turkey); Akkapulu N (Hacettepe University Hospital, Turkey); Karahan O, Damburaci N (Usak University Training and Research Hospital, Turkey); Hardwick R, Safranek P, Sujendran V, Bennett J, Afzal Z (Addenbrooke's Hospital, Cambridge, United Kingdom (UK)); Shrotri M, Chan B, Exarchou K, Gilbert T (Aintree University Hospital, Liverpool, UK); Amalesh T, Mukherjee D, Mukherjee S, Wiggins TH (Barking Havering and Redbridge NHS Trust, UK); Kennedy R, McCain S, Harris A, Dobson G (Belfast City Hospital, UK); Davies N, Wilson I, Mayo D, Bennett D (Royal Bournemouth Hospital, UK); Young R, Manby P (Bradford Royal Infirmary, UK); Blencowe N, Schiller M, Byrne B (University Hospitals Bristol NHS Foundation Trust, UK); Mitton D, Wong V, Elshaer A, Cowen M (Castle Hill Hospital, UK); Menon V, Tan LC, McLaughlin E, Koshy R (University Hospital of Coventry, UK); Sharp C (University Hospital Crosshouse, UK); Brewer H, Das N, Cox M, Al Khyatt W, Worku D (Royal Derby Hospital, UK); Iqbal R, Walls L, McGregor R (Edinburgh Royal Infirmary, UK); Fullarton G, Macdonald A, MacKay C, Craig C (Glasgow Royal Infirmary, UK); Dwerryhouse S, Hornby S, Jaunoo S, Wadley M (Gloucester Royal Hospital, UK); Baker C, Saad M, Kelly M, Davies A, Di Maggio F (Guy's and St Thomas's Hospitals, UK); McKay S, Mistry P, Singhal R, Tucker O, Kapoulas S, Powell-Brett S (Heartlands Hospital, UK); Davis P, Bromley G, Watson L (James Cook University Hospital, UK); Verma R, Ward J, Shetty V, Ball C, Pursnani K (Lancashire Teaching Hospitals NHS Foundation Trust, UK); Sarela A, Sue Ling H, Mehta S, Hayden J, To N (Leeds Teaching Hospitals NHS Trust, UK); Palser T, Hunter D, Supramaniam K, Butt Z, Ahmed A (University Hospitals of Leicester NHS Trust, UK); Kumar S, Chaudry A, Moussa O (Royal Marsden Hospital, UK); Kordzadeh A, Lorenzi B (Mid and South Essex NHS Foundation Trust, UK) Wilson M, Patil P, Noaman I (Ninewells Hospital, UK); Willem J (Norfolk and Norwich University Hospital); Bouras G, Evans R, Singh M, Warrilow H, Ahmad A (University Hospital of North Midlands, UK); Tewari N, Yanni F, Couch J, Theophilidou E, Reilly JJ, Singh P (Nottingham University Hospital, UK); van Boxel Gijs, Akbari K, Zanotti D, Sgromo B (Oxford University Hospitals); Sanders G, Wheatley T, Ariyarathenam A, Reece-Smith A, Humphreys L (Plymouth Hospitals NHS Trust, UK); Choh C, Carter N, Knight B, Pucher P (Queen Alexandra Hospital, Portsmouth, UK); Athanasiou A, Mohamed I, Tan B, Abdulrahman M (Queen Elizabeth Hospital Birmingham, UK); Vickers J, Akhtar K, Chaparala R, Brown R, Alasmar MMA (Salford Royal Foundation Trust, UK); Ackroyd R, Patel K, Tamhankar A, Wyman A (Sheffield Teaching Hospitals NHS Foundation Trust, UK); Walker R, Grace B (University Hospital Southampton NHS Foundation Trust, UK); Abbassi N, Slim N, Ioannidi L (University College Hospital, UK); Blackshaw G, Havard T, Escofet X, Powell A (University Hospital of Wales, UK); Owera A, Rashid F, Jambulingam P, Padickakudi J (Watford General Hospital, UK); Ben-Younes H, Mccormack K (University Hospital Wishaw, UK); Makey IA (Mayo Clinic in Florida, United States of America (USA)); Karush MK, Seder CW, Liptay MJ, Chmielewski G (Rush University Medical Center, USA); Rosato EL, Berger AC, Zheng R, Okolo E (Thomas Jefferson University, USA); Singh A, Scott CD, Weyant MJ, Mitchell JD (University of Colorado Hospital, USA).
